# Aspartoacylase suppresses prostate cancer progression by blocking LYN activation

**DOI:** 10.1186/s40779-023-00460-0

**Published:** 2023-06-05

**Authors:** Hong Weng, Kang-Ping Xiong, Wang Wang, Kai-Yu Qian, Shuai Yuan, Gang Wang, Fang Yu, Jun Luo, Meng-Xin Lu, Zhong-Hua Yang, Tao Liu, Xing Huang, Hang Zheng, Xing-Huan Wang

**Affiliations:** 1 Department of Urology, Zhongnan Hospital of Wuhan University, No. 169 Donghu Road, Wuchang District, Wuhan, 430071 China; 2 Center for Evidence-Based and Translational Medicine, Zhongnan Hospital of Wuhan University, Wuhan, 430071 China; 3 Department of Biological Repositories, Zhongnan Hospital of Wuhan University, Wuhan, 430071 China; 4 Hubei Key Laboratory of Tumor Biological Behaviors, Wuhan, 430071 China; 5 Department of Pathology, Zhongnan Hospital of Wuhan University, Wuhan, 430071 China; 6 Center for Pathology and Molecular Diagnostics, Wuhan University, Wuhan, 430071 China; 7 Wuhan Research Center for Infectious Diseases and Cancer, Chinese Academy of Medical Sciences, Wuhan, 430071 China

**Keywords:** Prostate cancer, Aspartoacylase, LYN, JNK, AP-1, C-Jun, Phosphorylation

## Abstract

**Background:**

Globally, despite prostate cancer (PCa) representing second most prevalent malignancy in male, the precise molecular mechanisms implicated in its pathogenesis remain unclear. Consequently, elucidating the key molecular regulators that govern disease progression could substantially contribute to the establishment of novel therapeutic strategies, ultimately advancing the management of PCa.

**Methods:**

A total of 49 PCa tissues and 43 adjacent normal tissues were collected from January 2017 to December 2021 at Zhongnan Hospital of Wuhan University. The advanced transcriptomic methodologies were employed to identify differentially expressed mRNAs in PCa. The expression of aspartoacylase (ASPA) in PCa was thoroughly evaluated using quantitative real-time PCR and Western blotting techniques. To elucidate the inhibitory role of ASPA in PCa cell proliferation and metastasis, a comprehensive set of in vitro and in vivo assays were conducted, including orthotopic and tumor-bearing mouse models (*n* = 8 for each group). A combination of experimental approaches, such as Western blotting, luciferase assays, immunoprecipitation assays, mass spectrometry, glutathione S-transferase pull-down experiments, and rescue studies, were employed to investigate the underlying molecular mechanisms of ASPA’s action in PCa. The Student’s* t*-test was employed to assess the statistical significance between two distinct groups, while one-way analysis of variance was utilized for comparisons involving more than two groups. A two-sided *P* value of less than 0.05 was deemed to indicate statistical significance.

**Results:**

ASPA was identified as a novel inhibitor of PCa progression. The expression of ASPA was found to be significantly down-regulated in PCa tissue samples, and its decreased expression was independently associated with patients’ prognosis (*HR* = 0.60, 95% CI 0.40–0.92, *P* = 0.018). Our experiments demonstrated that modulation of ASPA activity, either through gain- or loss-of-function, led to the suppression or enhancement of PCa cell proliferation, migration, and invasion, respectively. The inhibitory role of ASPA in PCa was further confirmed using orthotopic and tumor-bearing mouse models. Mechanistically, ASPA was shown to directly interact with the LYN and inhibit the phosphorylation of LYN as well as its downstream targets, JNK1/2 and C-Jun, in both PCa cells and mouse models, in an enzyme-independent manner. Importantly, the inhibition of LYN activation by bafetinib abrogated the promoting effect of *ASPA* knockdown on PCa progression in both in vitro and in vivo models. Moreover, we observed an inverse relationship between ASPA expression and LYN activity in clinical PCa samples, suggesting a potential regulatory role of ASPA in modulating LYN signaling.

**Conclusion:**

Our findings provide novel insights into the tumor-suppressive function of ASPA in PCa and highlight its potential as a prognostic biomarker and therapeutic target for the management of this malignancy.

**Supplementary Information:**

The online version contains supplementary material available at 10.1186/s40779-023-00460-0.

## Background

In 2020, a total of 1,414,259 novel prostate cancer (PCa) cases were documented, accompanied by 375,304 PCa-associated mortalities, ranking the second most prevalent malignancy in the male population globally [1]. Patients presenting low- or intermediate-risk localized disease exhibit favorable prognoses, as evidenced by a 10-year overall survival rate of 99%, contingent upon early detection and intervention [2, 3]. Conversely, once metastasis occurs, the 5-year survival rate for PCa plummets drastically to approximately 30% [4]. Moreover, the economic ramifications of PCa are substantial, with the cost of treatment escalating more precipitously for PCa than for any other cancer over the past decade [5].

Androgen deprivation therapy persists as the primary intervention for advanced PCa; however, the majority of hormone-sensitive malignancies ultimately progress to lethal castration-resistant PCa (CRPC) and/or metastatic disease [6]. PCa represents a complex, multifactorial condition characterized by intricate interactions among genetic, environmental, and lifestyle determinants [7, 8]. Despite extensive clinical and experimental investigations conducted over recent decades, the precise molecular mechanisms underpinning PCa pathogenesis remain unclear. Consequently, the identification of critical molecular regulators implicated in the disease progression would substantially contribute to the development of innovative therapeutic strategies for the management of patients with PCa.

Aminoacylases, comprising a family of cytosolic zinc-dependent metalloenzymes, are responsible for catalyzing the deacetylation of *N*-acyl-l-amino acids into acetate and free amino acids, playing a crucial role in protein synthesis [9, 10]. Aminoacylase 1 (ACY1) has been implicated as a putative tumor suppressor in a variety of carcinomas, including small-cell lung cancer [11], renal clear cell carcinoma [12], hepatocellular carcinoma [13], and neuroblastoma [10]. Another member of the aminoacylase family, aspartoacylase (ASPA, also referred to as ACY2), is an enzyme involved in the generation of acetate through the catalysis of *N*-acetyl-l-aspartate and has been associated with a severe childhood leukodystrophy known as Canavan disease, which originates from missense mutations [14]. Long et al. [10] employed bioinformatic data mining techniques to suggest that reduced expression of ACY1 or ASPA may be linked to unfavorable neuroblastoma prognosis. Nevertheless, the role of ASPA in the context of PCa has yet to be elucidated.

In this study, we utilized transcriptomic data from public datasets and our RNA-sequencing (RNA-Seq) dataset to identify potential regulators of PCa progression. Among several candidates, *ASPA* was selected for further investigation as it was the differentially expressed gene (DEG) most significantly associated with progression-free survival (PFS) in PCa, as validated by four Gene Expression Omnibus (GEO) datasets. To elucidate the potential role of ASPA in PCa progression, we employed gain- and loss-of-function approaches in vitro and in vivo to investigate ASPA as a suppressor of PCa progression and as a potential therapeutic target for human PCa.

## Methods

### Human tissue specimens

Prostate tissue samples were acquired from the patients who underwent prostatectomy from January 2017 through December 2021 at the Department of Urology, Zhongnan Hospital of Wuhan University. All participants were 18 years of age or older, and the study cohort consisted of 49 PCa tissue specimens and 43 adjacent normal tissue specimens. For RNA-Seq, 1 PCa sample with paired normal tissue, 17 PCa tissues, and 8 adjacent normal tissues were procured. Western blotting involved the collection of 18 PCa samples with paired normal tissues, while immunohistochemistry (IHC) employed 5 PCa samples with paired normal tissues. Furthermore, 9 PCa samples with paired normal tissues, 17 PCa tissues, and 14 adjacent normal tissues were gathered for RT-qPCR analysis. The demographic and clinical characteristics of the patients are presented in Additional file 1: Tables S1–S4. All specimens were independently and blindly evaluated by two expert pathologists. Prior to surgery, written informed consent was obtained from each participant or their designated representative. The study protocol was approved by the Medical Ethics Committee, Zhongnan Hospital of Wuhan University (2021125). A schematic outlining the experimental procedure was provided in Additional file 1: Fig. S1a.

### Cell cultures and treatments

The following cell lines, namely HEK293T, PC-3, and DU145, were procured from the Type Culture Collection of the Chinese Academy of Sciences (Shanghai, China): HEK293T and DU145 cells were cultivated in Dulbecco’s modified eagle medium (DMEM; SH30022.01, Gibco, USA), whereas PC-3 cells were maintained in RPMI-1640 medium (C11875500BT, Gibco, USA). The incubation conditions were maintained at 37 °C with a 5% CO_2_ atmosphere. The culture media were supplemented with 10% fetal bovine serum (FBS; A3160802, Gibco, USA) and 1% penicillin–streptomycin solution (15140-122, Gibco, USA). For transfection experiments, the HEK293T, PC-3, and DU145 cells were transfected with the designated plasmids utilizing the polyethyleneimine (PEI; 23966-1, Polysciences, USA) technique. With regard to cellular treatment, PC-3 cells underwent serum starvation in RPMI-1640 medium overnight, followed by treatment with JNK-IN-8 (1 μmol/L and 2 μmol/L, HY-13319, MCE, USA) and bafetinib (5 μmol/L, HY-50868, MCE, USA) for specified durations. All cultured cells were subjected to routine mycoplasma contamination screening via DNA detection method.

### Plasmid constructs and lentivirus transduction

In this study, the coding sequences of *ASPA*, *C-Jun*, *LYN*, *SRPK1*, *CSNK2A1*, *FYN*, and *GAK* were amplified from a human cDNA library via PCR utilizing primers delineated in Table 1. Concurrently, truncated or mutant sequences of ASPA and LYN were generated. Following amplification, overexpression PCR products were subcloned into pcDNA5 expression vectors. To achieve knockdown of the *ASPA* gene in PC-3 and DU145 cell lines, a lentiviral pLKO.1 vector was employed, as detailed in Table 2. HEK293T cells were transfected with the recombinant vector in conjunction with two packaging plasmids, pMD2.G and psPAX2, utilizing PEI transfection reagents procured from Polysciences (USA) for lentivirus production. Viral supernatants containing cell culture media were collected and purified 48 h post-transfection. Subsequently, the target cell lines underwent transduction for a period of 24 h with the lentiviral particles in the presence of 8 μg/mL polybrene. The successful selection of positively transduced cells was achieved through the application of 2 μg/mL puromycin.

**Table 1:** Primers for overexpression plasmids

Gene	Sequence 5′–3′	
ASPA	F	TCGGGTTTAAACGGATCCATGACTTCTTGTCACATTGCTG
	R	GGGCCCTCTAGACTCGAGCTAATGTAAACAGCAGCGAATAC
C-Jun	F	TCGGGTTTAAACGGATCCATGACTGCAAAGATGGAAACGACC
	R	GGGCCCTCTAGACTCGAGTCAAAATGTTTGCAACTGCTGC
LYN	F	TCGGGTTTAAACGGATCCATGGGATGTATAAAATCAAAAGGGAAAGACAGC
	R	GGGCCCTCTAGACTCGAGCTAAGGCTGCTGCTGGTATTGC
SRPK1	F	TCGGGTTTAAACGGATCCGCCACCATGGAGCGGAAAGTGCTTGC
	R	GGGCCCTCTAGACTCGAGGGAGTTAAGCCAAGGGTGCCG
CSNK2A1	F	TCGGGTTTAAACGGATCCGCCACCATGTCGGGACCCGTGCC
	R	GGGCCCTCTAGACTCGAGCTGCTGAGCGCCAGCGG
FYN	F	TCGGGTTTAAACGGATCCATGGGCTGTGTGCAATGTAAG
	R	GGGCCCTCTAGACTCGAGTTACAGGTTTTCACCAGGTTGGTAC
GAK	F	TCGGGTTTAAACGGATCCGCCACCATGTCGCTGCTGCAGTCG
	R	GGGCCCTCTAGACTCGAGGAAGAGGGGCCGGGAGC
LYN (1-230)	F	TCGGGTTTAAACGGATCCATGGGATGTATAAAATCAAAAGGGAAAGACAGC
	R	GGGCCCTCTAGACTCGAGCTACTTGGGACTAATACAAGCCTTCTCC
LYN (231-512)	F	TCGGGTTTAAACGGATCCCCACAGAAGCCATGGGATAAAGATGC
	R	GGGCCCTCTAGACTCGAGCTAAGGCTGCTGCTGGTATTGC
ASPA (1-212)	F	TCGGGTTTAAACGGATCCATGACTTCTTGTCACATTGCTG
	R	GGGCCCTCTAGACTCGAGCTATCCTTCATTGAAATGATGTATAAAATCAAGAG
ASPA (213-313)	F	TCGGGTTTAAACGGATCCAAAGAATTTCCTCCCTGCGC
	R	GGGCCCTCTAGACTCGAGCTAATGTAAACAGCAGCGAATAC

*ASPA* aspartoacylase*, C-Jun* v-Jun avian sarcoma virus 17 oncogene homolog, *FYN* tyrosine-protein kinase Fyn, *GAK* cyclin G associated kinase, *LYN* Lck/Yes-related novel protein tyrosine kinase, *SRPK1* serine/arginine-rich protein-specific kinase 1, *F* forward, *R* reverse

**Table 2:** Primers for lentiviral plasmids

Gene	Sequence 5′–3′	
shASPA-1	F	CCGGGCCAAGTATCCTGTGGGTATACTCGAGTATACCCACAGGATACTTGGCTTTTTG
	R	AATTCAAAAAGCCAAGTATCCTGTGGGTATACTCGAGTATACCCACAGGATACTTGGC
shASPA-2	F	CCGGGCTGCTATCATCCATCCTAATCTCGAGATTAGGATGGATGATAGCAGCTTTTTG
	R	AATTCAAAAAGCTGCTATCATCCATCCTAATCTCGAGATTAGGATGGATGATAGCAGC
shASPA-3	F	CCGGGGCGCTGAGATTCAGAGAACACTCGAGTGTTCTCTGAATCTCAGCGCCTTTTTG
	R	AATTCAAAAAGGCGCTGAGATTCAGAGAACACTCGAGTGTTCTCTGAATCTCAGCGCC

*ASPA* aspartoacylase,* F* forward, *R* reverse

### Cell counting kit 8 (CCK-8) assays

CCK-8 assays were conducted utilizing CCK-8 reagents (B34304, Bimake, USA) in accordance with the manufacturer’s protocol. Transfected cells were plated in 96-well plates (167008, Thermo Fisher Scientific, USA) at a density of 3000 cells per well. PCa cell proliferation was evaluated at 1, 2, 3, 4, and 5 days post-seeding. Subsequently, a 10% solution of CCK-8 was added to each well, followed by an incubation at 37 °C for an additional 2 h under controlled conditions. Optical density (OD) measurements were obtained at a wavelength of 450 nm to assess cell viability.

### Colony formation assays

In reference to colony formation assays, PCa cells were propagated in 6-well plates at a density of 2000 cells per well. The cells were subsequently incubated at 37 °C for a period of 14 days, with the culture medium replaced three times per week to ensure optimal growth condition. Following the incubation period, the discernible cell colonies within each well were exposed to a 0.1% crystal violet staining solution (CAS No. 548-62-9) for 15 min, facilitating visualization and enumeration of the colonies.

### 5-Ethynyl-2′-deoxyuridine (EdU) incorporation assays

For the EdU assay experiments, a total of 1 × 10^5^ cells were subjected to the standardized procedures outlined by the EdU assay kit. Subsequent to the incubation with EdU, the PCa cells were washed twice using PBS to remove residual reagents. The cells were then fixed with 200 μL of 4% paraformaldehyde solution (G1101-500ML, Service bio, China) for 30 min, followed by Apollo®567 fluorophore staining to visualize EdU incorporation. Concurrently, Hoechst 33342 dye (DAPI) was employed for the selective staining of nuclear DNA. Imaging of the stained samples was performed using a High Content Imaging Analysis System (Operetta CLS, PerkinElmer, USA), and the number of EdU-positive cells was quantified to assess cellular proliferation.

### Transwell assays

Transwell migration and invasion assays were employed to evaluate the migratory and invasive properties of PCa cells. These assays utilized Corning Transwell permeable supports (REF 3421, Corning, USA) with or without the addition of Matrigel (354248, Corning, USA) for the migration and invasion assays, respectively.

For the migration assay, 5 × 10^4^ PCa cells suspended in serum-free medium were seeded into the upper chambers containing inserts with an 8.0 μm pore size. The lower chambers were filled with 600 μL of complete medium. These chambers were then incubated at 37 °C in a humidified atmosphere containing 5% CO_2_ for 24 h. Following incubation, the cells that had migrated to the bottom surface of the membrane were fixed using 4% paraformaldehyde and stained with crystal violet. Non-migrated cells were removed from the upper membrane surface with a cotton swab. Migrated cells were visualized and counted using an ECLIPSE 80i optical microscope (Nikon, Japan) equipped with a 10× magnification lens, with eight random regions being analyzed to quantify the number of cells that had migrated through.

For the invasion assay, the Transwell polycarbonate membrane was coated with 5 μg of Matrigel Basement Membrane Matrix (354248, Corning, USA) to simulate an extracellular matrix. The subsequent steps followed the same protocol as described for the Transwell migration assay. The number of invading cells on the lower side of the membrane was determined and quantified as previously described.

### Quantitative reverse transcription PCR (RT-qPCR)

Total RNA was extracted from prostate tissue samples and cultured PCa cells utilizing TRIzol reagent (Invitrogen, USA) in accordance with the manufacturer's guidelines. The accumulation of PCR products was detected using SYBR Green PCR Master Mix (04887352001, Roche, Switzerland). To ensure accurate quantification, the relative mRNA expression levels of the target genes were normalized to the mean expression level of the housekeeping gene, *ACTB*. RT-qPCR was performed using a LightCycler 480 System (Roche, Switzerland) as per the established protocol. The primer sequences employed in this study can be found in Table 3.

**Table 3:** Primers for RT-qPCR detection

Gene	Sequence 5′–3′	
*ASPA*	F	CAGCCTCAAGGGGTTCTGAG
	R	ATAGACCTCAATGGCGCAGG
*CCND1*	F	CAGATCATCCGCAAACACGC
	R	AGGCGGTAGTAGGACAGGAA
*PCNA*	F	CACTCCACTCTCTTCAACGGT
	R	ATCCTCGATCTTGGGAGCCA
*MYC*	F	TCGGGTAGTGGAAAACCAGC
	R	TCCTCCTCGTCGCAGTAGAA
*CDH1*	F	TGGGCCAGGAAATCACATCC
	R	GGCACCAGTGTCCGGATTAA
*CDH2*	F	AGTGGCAGCTGGACTTGATC
	R	CCGTGGCTGTGTTTGAAAGG
*MMP9*	F	TTTGAGTCCGGTGGACGATG
	R	TTGTCGGCGATAAGGAAGGG
*ACTB*	F	CATGTACGTTGCTATCCAGGC
	R	CTCCTTAATGTCACGCACGAT

*ASPA* aspartoacylase*, ACTB* actin beta, *CCND1* cyclin D1, *CDH1* cadherin 1, *CDH2* cadherin 2, *PCNA* proliferating cell nuclear antigen, *MYC* v-Myc myelocytomatosis viral oncogene homolog, *MMP9* matrix metallopeptidase 9, *F* forward, *R* reverse

### Western blotting

The total proteins from tissues or cells were extracted using RIPA lysis buffer containing protease and phosphatase inhibitors (04693132001 and 4906837001, respectively; Roche, Switzerland). The lysates were sonicated for 5 min at 40 amp using a Qsonica Sonication System. Then, the sonicated lysates were centrifuged in a microfuge at 12,000 r/min at 4 °C for 10 min to eliminate the precipitates. Bicinchoninic acid assay (BCA) Protein Assay Kit (Thermo Fisher Scientific, USA) quantified the total proteins. On 10% SDS-PAGE gels, the protein samples were loaded and separated before being transferred to PVDF membranes (Millipore, USA). The membranes were then blocked with 5% skim milk in TBST at room temperature for 60 min, incubated with primary antibodies at 4 °C overnight, and then with the appropriate horseradish peroxidase-conjugated secondary antibodies. ECL Western blotting Substrate kit (Thermo Fisher Scientific, USA) was used to detect the signals, and the ChemiDoc MP Imaging System (Bio-Rad, USA) was used to visualize them. The antibodies were presented in Table 4.

**Table 4:** Antibodies used in the present study

Antibodies	Source	Identifier
Anti-ASPA, dilution: 1:1000	Proteintech	Cat# 13244-1-AP, RRID: AB_2274358
Anti-PCNA, dilution: 1:1000	Proteintech	Cat# 10205-2-AP, RRID: AB_2160330
Anti-Cyclin D1, dilution: 1:1000	GeneTex	Cat# GTX16663, RRID: AB_422349
Anti-N-Cadherin, dilution: 1:1000	ABclonal	Cat# A3045, RRID: AB_2863024
Anti-E-Cadherin, dilution: 1:1000	ABclonal	Cat# A11492, RRID: AB_2758582
Anti-MMP9, dilution: 1:1000	ABclonal	Cat# A0289, RRID: AB_2757101
Anti-Ki67, dilution: 1:1000	Santa Cruz Biotechnology	Cat# sc-7846, RRID: AB_2142374
Anti-p-C-Jun (Ser73), dilution: 1:1000	Cell Signaling Technology	clone D47G9, Cat# 3270, RRID: AB_2129575
Anti-C-Jun, dilution: 1:1000	Cell Signaling Technology	clone 60A8, Cat# 9165, RRID: AB_2130165
Anti-p-C-Fos (Ser32), dilution: 1:1000	Cell Signaling Technology	clone D82C12, Cat# 5348, RRID: AB_105571095384
Anti-C-Fos, dilution: 1:1000	Cell Signaling Technology	clone 9F6, Cat# 2250, RRID: AB_2247211
Anti-p-JNK (Thr183/Tyr185), dilution: 1:1000	Cell Signaling Technology	clone 81E11, Cat# 4668, RRID: AB_823588
Anti-JNK, dil: 1:1000	Cell Signaling Technology	Cat# 9252, RRID: AB_2250373
Anti-p-P38 (Thr180/Tyr182), dilution: 1:1000	Cell Signaling Technology	clone D3F9, Cat# 4511, RRID: AB_2139682
Anti-P38, dilution: 1:1000	ABclonal	Cat# A14401, RRID: AB_2761271
Anti-p-ERK1/2 (Thr202/Tyr204), dilution: 1:1000	Cell Signaling Technology	clone D13.14.4E, Cat# 4370, RRID: AB_2315112
Anti-ERK1/2, dilution: 1:1000	Cell Signaling Technology	clone 137F5, Cat# 4695, RRID: AB_390779
Anti-p-LYN (Y396), dilution: 1:1000	ABclonal	Cat# AP1050, RRID: AB_2863923
Anti-LYN, dilution: 1:1000	ABclonal	Cat# A2093, RRID: AB_2764113
Anti-β-actin, dilution: 1:50,000	ABclonal	Cat# AC026, RRID: AB_2768234
Anti-HA, dilution: 1:10,000	MBL International	Cat# M180-3, RRID: AB_10951811
Anti-Flag, dilution: 1:10,000	MBL International	Cat# M185-3LL, RRID: AB_11126775

*ASPA* aspartoacylase*, C-Jun* v-Jun avian sarcoma virus 17 oncogene homolog, *C-Fos* v-Fos FBJ murine osteosarcoma viral oncogene homolog, *ERK* extracellular regulated protein kinases, *JNK* c-Jun N-terminal kinase, *PCNA* proliferating cell nuclear antigen, *MMP9* matrix metallopeptidase 9, *LYN* Lck/Yes-related novel protein tyrosine kinase, *MBL* mannose/mannan-binding lectin, *RRID* research resource identifiers

### Immunoprecipitation (IP) assay

For the coimmunoprecipitation (Co-IP) assays, HEK293T and PC-3 cells were cotransfected with the designated plasmids and subsequently harvested in cold IP lysis buffer containing protease and phosphatase inhibitors after a 24 h incubation period. The cell lysates were centrifuged, resulting in a protein-rich supernatant that was subjected to IP using protein G agarose beads. This mixture was incubated overnight at 4 °C with specified anti-tag antibodies. To perform endogenous Co-IP, PC-3 cells were lysed in IP buffer and immunoprecipitated with appropriate primary antibodies. Subsequently, the beads were thoroughly washed with lysis buffer, resuspended in 2 × SDS loading buffer, and heated to boiling. The samples were then analyzed through SDS-PAGE to determine protein interactions.

### Glutathione S-transferase (GST) pull-down assays

HEK293T cells were seeded onto 6-well plates and subsequently transfected with designated plasmids for a period of 24 h, allowing for the expression of the desired GST-fusion proteins, which were to be employed in the GST pull-down assay. Following the transfection period, cells were harvested by administering 500 µL of lysis buffer, enriched with a protease inhibitor cocktail (04693132001, Roche, Switzerland) to each well. The resulting cell lysates were then incubated with either GST or GST-fused protein immobilized on glutathione-Sepharose 4B beads at 4 °C, utilizing end-over-end rotation for an overnight duration. Subsequent to the incubation, the beads were thoroughly washed with low-salt GST buffer on three separate occasions, and subsequently heated for 10 min in the presence of 2 × SDS loading buffer. Lastly, the samples were subjected to immunoblot analysis, employing the appropriate primary antibodies to detect the proteins of interest.

### RNA-seq and bioinformatic analysis

Total RNA was extracted from 18 PCa and 9 normal tissue samples for RNA-seq analysis using the MGISEQ 2000 platform. Raw sequencing reads were aligned to the Ensembl Human (GRCh38/hg38) reference genome using the HISAT2 software. Sequencing counts of The Cancer Genome Atlas Prostate Adenocarcinoma (TCGA-PRAD) dataset, encompassing 498 PCa and 52 normal tissues, were retrieved from the TCGA database (https://portal.gdc.cancer.gov/). DEGs were determined based on a fold change greater than 2 and an adjusted *P* value of less than 0.05. The “ggplot2” *R* package facilitated the creation of a volcano plot illustrating fold changes and *P* values for all genes. To identify critical genes potentially associated with PCa progression, the overlapping DEGs in the top 5% of TCGA-PRAD and RNA-seq datasets were ascertained. Subsequently, the influence of these overlapping DEGs on PFS was evaluated utilizing the “survival” and “survminer” *R* packages, employing the log-rank test. The associations between PFS-related overlapping DEGs and clinical phenotypes of PCa were also investigated. Validation of the PFS-related overlapping DEGs was carried out using raw data acquired from the GEO database (https://www.ncbi.nlm.nih.gov/geo/), including the GSE62872 [15, 16], GSE88808 [17], GSE70768 [18], and GSE32571 [19] datasets. The German Cancer Research Center-Deutsches Krebsforschungszentrum (DKFZ) Cancer Cell 2018 [20] and Memorial Sloan Kettering Cancer Center (MSKCC) Cancer Cell 2010 [21] datasets were accessed via cBioPortal (https://www.cbioportal.org/) [22]. The Genotype-Tissue Expression project (GTEx) dataset was obtained from the GTEx database (https://www.gtexportal.org/home/datasets).

### Hierarchical clustering analysis

In the process of assessing the congruence among discrete specimens, hierarchical clustering engenders a stratified, nested clustering dendrogram. Utilizing the Unweighted Pair Group Method with Arithmetic Mean (UPGMA) approach facilitated the execution of hierarchical clustering analyses. To visualize the outcomes, the “hclust” function, incorporated within the “stats” *R* package, was employed. The inter-group distance was determined by adhering to the default algorithm, and the hierarchical clustering was conducted on the basis of the mRNA expression matrix. This methodology accentuated the disparities between the two groups under investigation.

### Gene set enrichment analysis (GSEA)

In the GSEA, the gene sets were derived from the Molecular Signatures Database (MsigDB) hallmark and C2 curated datasets. These gene sets were arranged according to the degree of differential expression. Subsequently, the concentration of the gene sets within the ranking table was scrutinized. The analysis was conducted utilizing GSEA v3.0 software with the “Signal2Noise” metric criteria on the Java platform [23, 24]. Statistically significant gene sets were identified based on a nominal *P* value less than 0.05 and a false discovery rate (FDR) less than 0.25. The nominal *P* value was determined through an empirical phenotype-based permutation test.

### Luciferase reporter assays

The Dual-Luciferase Reporter Assay System kit (E1910, Promega, USA) was employed to investigate luciferase activity in accordance with the manufacturer’s guidelines. For these luciferase reporter assays, PC-3 cells underwent transfection utilizing Lipofectamine 2000 (11668030, Thermo Fisher Scientific, USA) in conjunction with the specified plasmid constructs, including ACTB, 45 pathways (courtesy of Pro. Xiao-Dong Zhang in the Wuhan University), ASPA overexpression, and *ASPA* knockdown. Subsequent to a 24 h period post-transfection, the cells were harvested and subjected to lysis in 100 μL 1 × Passive Lysis Buffer (Promega, USA) through vigorous agitation for 30 min. A 10 μL aliquot of the resultant lysate was then utilized for the luciferase assays, conducted on a GloMax 20/20 Luminometer (Promega, USA). Firefly luciferase activity was subsequently normalized to Renilla luciferase activity for data analysis.

### Mass spectrometry analysis

Flag and Flag-ASPA were transfected into human PC-3 cells and incubated for 48 h. Subsequently, the cells were lysed employing an IP buffer, and Flag-ASPA was immunoprecipitated following the previously established protocol for the IP assay. The obtained IP samples were subjected to SDS-PAGE and run for a limited distance (0.5 cm) based on the migration of the bromophenol blue dye. In-gel proteins were reduced utilizing dithiothreitol for 30 min and subsequently alkylated with iodoacetamide for 45 min at room temperature under dark condition. The in-gel proteins were then digested with trypsin enzymes at 37 °C overnight. Peptide mixtures were extracted from the gel via incubation in a 60% ACN/0.1% TFA solution, performed three times. Following this, the peptide mixtures were dissolved in buffer A and loaded onto a reversed-phase trap column (Acclaim PepMap100, Thermo Fisher Scientific, USA). The samples were then connected to a C18-reversed-phase analytical column (Easy Column, Thermo Fisher Scientific, USA) and separated using a linear gradient of buffer B at a constant flow rate of 400 nL/min. A Q-Exactive mass spectrometer (Thermo Fisher Scientific, USA) was coupled to an Easy nLC system (Proxeon Biosystems, Denmark) for mass spectrometry analysis.

### Animal models

Mice (*n* = 96) were maintained and propagated in a specific pathogen-free, temperature-regulated environment. To establish subcutaneous tumor-bearing mouse models, male BALB/c nude mice aged 5–6 weeks were randomized into control group (*n* = 8) and ASPA group (*n* = 8) or shRNA group (*n* = 8) and shASPA group (*n* = 8) and were inoculated with 5 × 10^6^ corresponding PC-3 cells suspended in 200 μL PBS per mouse. Tumor formation was monitored, and tumor volumes were quantified using a Vernier caliper. After 32 days, mice were euthanized, and tumor tissues were harvested and weighed. Tumor volumes were determined by the formula 0.5 × long diameter × short diameter × short diameter.

For orthotopic xenograft tumor-based experimental models, male BALB/c nude mice aged 5–6 weeks were randomized into control group (*n* = 8) and ASPA group (*n* = 8) or shRNA group (*n* = 8) and shASPA group (*n* = 8) and were immobilized using surgical tape and anesthetized with isoflurane. The prostate was exposed under a microscope, and 1 × 10^6^ corresponding PC-3 cells suspended in 30 μL PBS were injected per mouse. Post-injection, the needle inlet was clamped with iris forceps and sealed using a drop of 3 M tissue glue. Upon glue solidification, the iris forceps were removed, and the anatomical positions of the organs were restored. Mice were returned to their cages following recovery. After 30 days, mice were euthanized, and tumor tissues were harvested and weighed.

For rescue assays, nude mice were randomized into shRNA + vehicle group (*n* = 8), shASPA + vehicle group (*n* = 8), shRNA + bafetinib group (*n* = 8) and shASPA + bafetinib group (*n* = 8). Mice were subcutaneously injected with corresponding PC-3 cells. Upon reaching an average tumor volume of 100 mm^3^, mice were administered 20 mg/kg bafetinib (CAS859212-16-1, T6311, Topscience, USA) via oral gavage daily, while corn oil served as a control. Mice were sacrificed after 13 days, and tumor tissues were harvested and weighed. All animal experiments were approved by the Animal Care and Use Committee, Wuhan University Zhongnan Hospital (ZN2021113).

### IHC

IHC was conducted employing a PV-9001 kit (ZSGB-Bio, China) following the manufacturer’s recommended protocols. Upon dewaxing and hydration, tissue sections were immersed in an EDTA buffer (pH 9.0) and subjected to antigen retrieval through heating in a pressure cooker for 20 min. The sections were subsequently cooled to room temperature in EDTA buffer and rinsed in PBS for three cycles, each lasting 3 min. Thereafter, the sections were treated with a 3% hydrogen peroxide solution at room temperature for 20 min and rinsed in PBS buffer for three cycles, each lasting 3 min. Blocking was performed using 10% bovine serum albumin (BSA; NA8692, Bomei, China) for 30 min at 37 °C. The paraffin-embedded sections were incubated with primary antibodies targeting ASPA (1:200, 13244-1-AP, RRID: AB_2274358, Proteintech, USA) or Ki67 (1:100, GB13030-M-2, Servicebio, China) at 4 °C overnight, followed by rinsing in PBS buffer for three cycles, each lasting 3 min. The sections were subsequently incubated with reaction enhancers (reagent 2, PV-9001, ZSGB-Bio, China) for 30 min at 37 °C and washed in PBS buffer for three cycles, each lasting 3 min. The paraffin sections were then exposed to an enhanced enzyme-labeled goat anti-rabbit IgG polymer (reagent 3, PV-9001, ZSGB-Bio, China) for 20 min at room temperature and washed in PBS buffer for three cycles, each lasting 3 min. Visualization of the paraffin sections was achieved through the application of DAB (ZLI-9018, ZSGB-Bio, China), followed by counterstaining with hematoxylin (G1004, Servicebio, China). Finally, the paraffin sections were cover-slipped and subjected to microscopic examination utilizing a light microscope (ECLIPSE 80i, Nikon, Japan).

### Statistical analysis

The continuous data were expressed as mean ± standard deviation (SD) derived from a minimum of three independent experiments. To assess the statistical significance between two distinct groups, Student’s* t*-test was employed, while one-way analysis of variance (ANOVA) was utilized for comparisons involving more than two groups. A two-sided *P* value of less than 0.05 was deemed to indicate statistical significance. All statistical analyses were performed using GraphPad Prism version 9.0 (GraphPad Software, USA) or R software (version R-4.2.1).

## Results

### ASPA is down-regulated in PCa

**Fig. 1:**
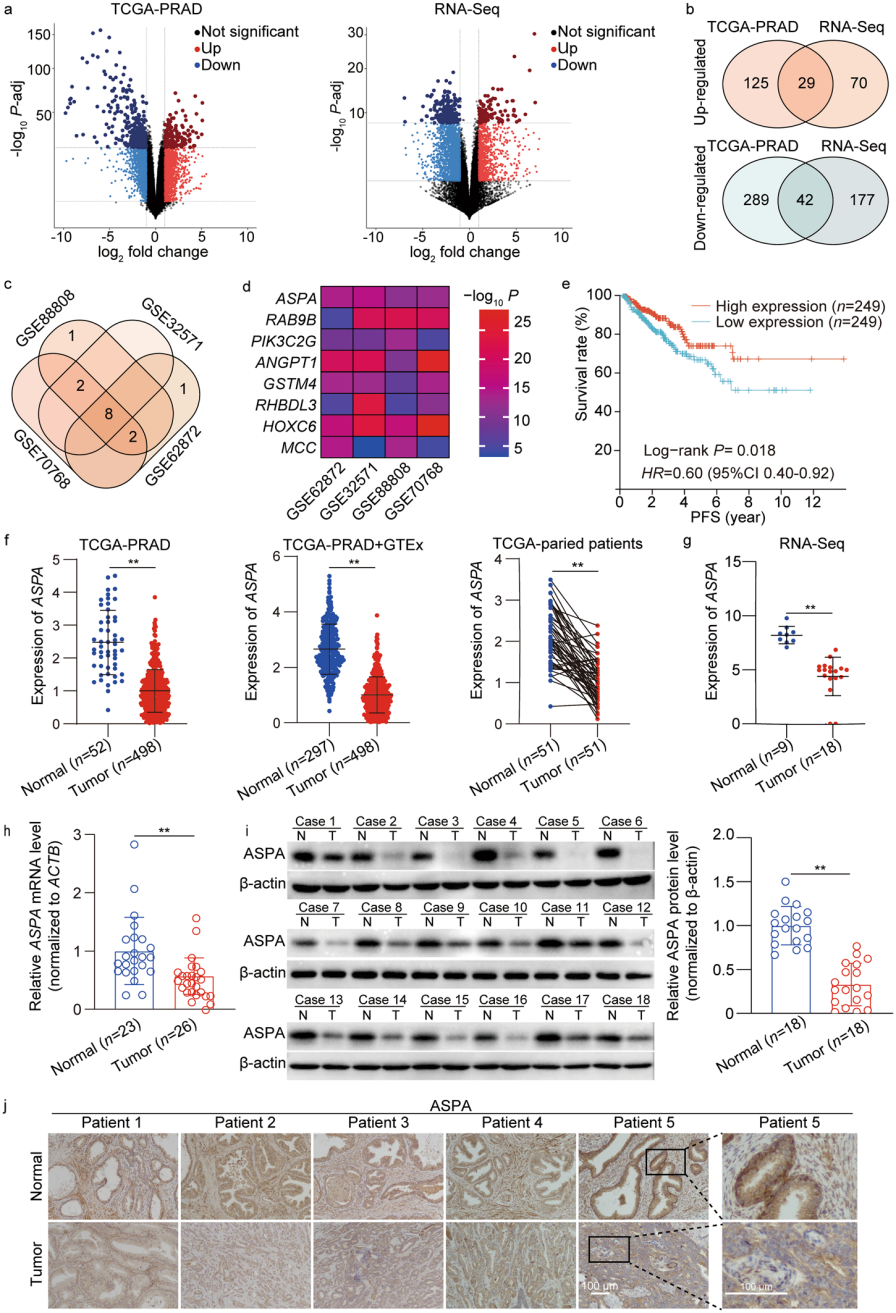
ASPA is down-regulated in PCa. **a** Volcano plot showed all expression changes of genes in the TCGA-PRAD dataset including 498 PCa and 52 normal tissues (left) and our RNA-seq dataset including 18 PCa and 9 normal tissues (right). Blue and red dots indicate down- and up-regulated genes, respectively. **b** Venn diagram showed the overlapping differentially expressed genes (DEGs) in TCGA-PRAD and RNA-Seq datasets. **c** Venn diagram showed the overlap of PFS-related DEGs in four GEO datasets. **d** Heatmap analysis of the overlapping PFS-related DEGs between PCa and normal tissues in four GEO datasets. **e** Kaplan–Meier curves of *ASPA* in PCa for PFS. **f** The expression level of *ASPA* in PCa from TCGA-PRAD and GTEx datasets (left) and in paired tissues from patients of TCGA-PRAD (right). **g** The expression level of *ASPA* in 9 normal prostate tissues and 18 PCa tissues from RNA-Seq dataset. **h** The expression level of *ASPA* in normal tissues (*n* = 23) and PCa (*n* = 26) by RT-qPCR. The mRNA expression levels were normalized to ACTB levels. **i** Representative Western blotting analysis (left) and quantification results (right) of ASPA expression in 18 PCa samples with paired normal tissues. Protein expression was normalized to β-actin levels. **j** Representative immunohistochemical staining images of ASPA expression in 5 PCa samples with paired normal tissues (scale bar = 100 μm). The data are presented as mean ± standard deviation (SD). ASPA aspartoacylase, GEO gene expression omnibus, GTEx Genotype-Tissue Expression project, HR hazard ration, CI confidence interval, TCGA-PRAD The Cancer Genome Atlas Prostate Adenocarcinoma, RNA-Seq RNA sequencing, TCGA The Cancer Genome Atlas, PFS progression-free survival, RT-qPCR real-time quantitative PCR, PCa prostate cancer, N normal, T tumor. **P < 0.01

A noteworthy observation was the identification of 29 up-regulated and 42 down-regulated overlapping DEGs within the top 5% of DEGs derived from TCGA-PRAD and RNA-Seq datasets (Fig. 1a, b). Among these genes, 8 highly expressed genes associated with low PFS in the up-regulated category and 25 lowly expressed genes associated with low PFS in the down-regulated category were discerned.

The association between the 33 identified PFS-related DEGs and pathological T stage, N stage, and Gleason score in TCGA-PRAD dataset was assessed (Additional file 1: Fig. S1b). Subsequent validation of the PFS-related DEGs was conducted using four GEO datasets, revealing *ASPA* as the most DEG among them (Fig. 1c, d). Kaplan–Meier survival analysis indicated that PCa patients with elevated *ASPA* expression exhibited improved PFS (*HR* = 0.60, 95%CI 0.40–0.92, *P* = 0.018; Fig. 1e).

The analysis further demonstrated that *ASPA* mRNA levels were suppressed in PCa samples compared to normal prostate specimens across public and RNA-Seq datasets (*P* < 0.01; Fig. 1f, g; Additional file 1: Fig. S1c). Additionally, a negative correlation was observed between *ASPA* expression and pathological T stage, N stage, Gleason score, and biochemical recurrence (BCR) (*P* < 0.01, Additional file 1: Fig. S1d). A positive correlation was detected between *ASPA* expression and the expression of *PTEN* (Spearman rho = 0.38, *P* < 0.01 in TCGA-PRAD dataset; Spearman rho = 0.53, *P* < 0.01 in DKFZ Cancer Cell 2018 dataset; Spearman rho = 0.36, *P* < 0.01 in MSKCC Cancer Cell 2010 dataset; Additional file 1: Fig. S1e) and *SPOP* (Spearman rho = 0.45, *P* < 0.01 in TCGA-PRAD dataset; Spearman rho = 0.31, *P* < 0.01 in DKFZ Cancer Cell 2018 dataset; Spearman rho = 0.38, *P* < 0.01 in MSKCC Cancer Cell 2010 dataset; Additional file 1: Fig. S1e).

Lastly, the RT-qPCR analysis of clinical specimens revealed a down-regulation of *ASPA* mRNA levels in PCa patients compared to normal tissue samples (*P* < 0.01; Fig. 1h). Western blotting substantiated a significant decrease in ASPA protein levels in PCa tissues relative to adjacent samples (*P* < 0.01; Fig. 1i), corroborating the reduction in *ASPA* mRNA levels. IHC assays displayed analogous findings (Fig. 1j).

### ASPA overexpression inhibits the proliferation and migration of PCa cells in vitro

**Fig. 2:**
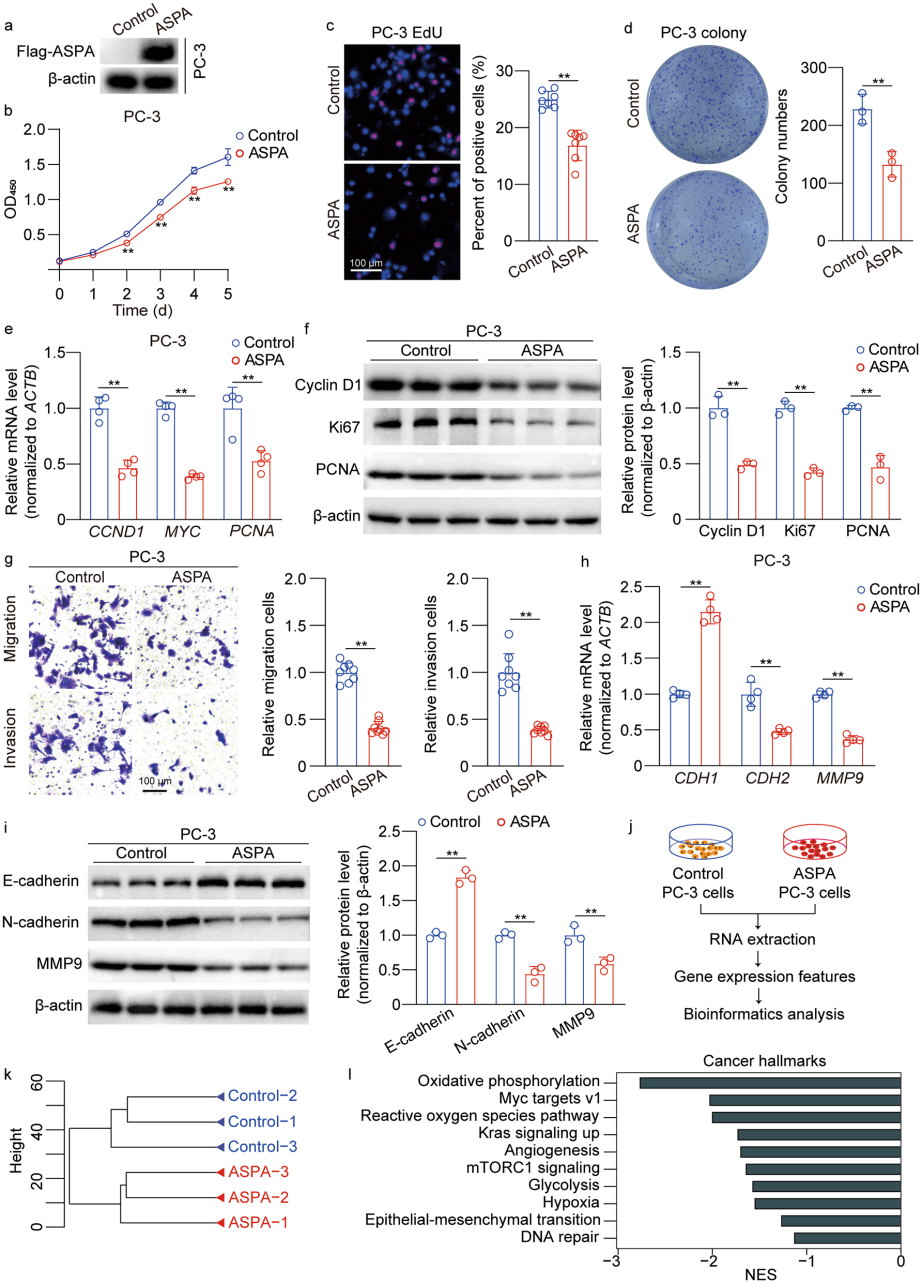
ASPA overexpression inhibits PC-3 cell proliferation and migration in vitro. **a** Western blotting results for ASPA protein expression in PC-3 cells transfected with control or ASPA overexpression vector. Protein expression levels were normalized to β-actin levels. **b** CCK-8 assay showed that ASPA overexpression inhibited PC-3 cell proliferation. **c** Representative images of EdU-positive PC-3 cells transfected with control or ASPA overexpression vector (scale bar = 100 μm). The graph on the right shows the percentage of EdU-positive nuclei. The data were obtained from 7 fields of 3 independent experiments. **d** The colony formation assay showed that ASPA overexpression inhibited PC-3 colony formation. The graph on the right shows the colony numbers from 3 independent experiments. **e** RT-qPCR results of proliferation-related genes in PC-3 cells transfected with control or ASPA overexpression vectors. The mRNA expression levels were normalized to *ACTB* levels. **f** Western blotting results (left) and quantification results (right) for proliferation-related proteins in PC-3 cells transfected with control or ASPA overexpression vector. Protein expression levels were normalized to β-actin levels. **g** Transwell assays showed that ASPA overexpression inhibited PC-3 cell migration and invasion (scale bar = 100 μm). The graph on the right shows the migrating cells and the invading cells. The data were obtained from 8 fields of 3 independent experiments. **h** RT-qPCR results of epithelial–mesenchymal transition genes in PC-3 cells transfected with control or ASPA overexpression vector. The mRNA expression levels were normalized to *ACTB* levels. **i** Western blotting results (left) and quantification results (right) for epithelial–mesenchymal transition proteins in PC-3 cells transfected with control or ASPA overexpression vector. Protein expression levels were normalized to β-actin levels. **j** Flow chart of RNA-Seq in PC-3 cells transfected with control or ASPA overexpression vector. **k** Cluster analysis showed the global sample distribution profiles of the control group and ASPA overexpression group based on the RNA-Seq dataset in PC-3 cells. **l** GSEA results showed significantly altered cancer hallmarks based on the dataset of RNA-Seq in PC-3 cells from the control group and ASPA overexpression group. The data are presented as the mean ± standard deviation (SD). ASPA aspartoacylase, CCK-8 cell counting kit 8, CCND1 cyclin D1, CDH1 cadherin 1, CDH2 cadherin 2, EdU 5-ethynyl-2′-deoxyuridine, GSEA gene set enrichment analysis, MYC v-Myc myelocytomatosis viral oncogene homolog, MMP9 matrix metallopeptidase 9, NES normalized enrichment score, OD optical density, PCNA proliferating cell nuclear antigen, RNA-Seq RNA sequencing, RT-qPCR real-time quantitative PCR. **P < 0.01

The overexpression of ASPA was confirmed through Western blotting analysis (Fig. 2a, Additional file 1: Fig. S2a). Our findings demonstrated a significant inhibition of PCa cell proliferation due to ASPA overexpression (*P* < 0.01; Fig. 2b, Additional file 1: Fig. S2b). This suppressive effect of ASPA on PCa cells was further corroborated by EdU assays (*P* < 0.01; Fig. 2c, Additional file 1: Fig. S2c) and colony formation assays (*P* < 0.01; Fig. 2d, Additional file 1: Fig. S2d). In accordance with the cell proliferation and viability assays, RT-qPCR analysis revealed a suppression of mRNA levels of *CCND*1, *MYC*, and *PCNA* upon ASPA overexpression (*P* < 0.01; Fig. 2e, Additional file 1: Fig. S2e). Concurrently, Western blotting analysis indicated an inhibition of cyclin D1, Ki67, and PCNA expression due to ASPA overexpression (*P* < 0.01; Fig. 2f, Additional file 1: Fig. S2f).

We utilized Transwell assays to provide evidence that the up-regulation of ASPA expression results in the inhibition of migration and invasion capabilities of PCa cells (*P* < 0.01; Fig. 2g, Additional file 1: Fig. S2g). Additionally, our findings indicate that overexpression of ASPA contributes to a decrease in *CDH2* and *MMP9* expression levels, concomitant with an elevation in *CDH1* expression levels (*P* < 0.05 or *P* < 0.01; Fig. 2h; Additional file 1: Fig. S2h). In a complementary approach, Western blotting analyses were employed to demonstrate that ASPA overexpression led to a reduction in N-cadherin and MMP9 expression, while simultaneously promoting the expression of E-cadherin (*P* < 0.01; Fig. 2i; Additional file 1: Fig. S2i).

Unsupervised hierarchical clustering distinctly separated the samples into two subclusters (Fig. 2j, k). GSEA and heatmaps indicated that cellular signaling pathways and genes associated with oxidative phosphorylation, Myc targets v1, reactive oxygen species pathway, Kras signaling up, angiogenesis, mTORC1 signaling, glycolysis, hypoxia, epithelial–mesenchymal transition, and DNA repair were significantly down-regulated upon ASPA overexpression (*P* < 0.05 for *SOD2* and *CSF2*; *P* < 0.01 for *COX6C*, *BDH2*, *UQCRH*, *ODC1*, *PGK1*, *RPL22*, *CAT*, *G6PD*, *CCL20*, and *SLP1*; Fig. 2l, Additional file 1: Fig. S2j-l).

### *ASPA* knockdown promotes the proliferation and migration of PCa cells in vitro

**Fig. 3:**
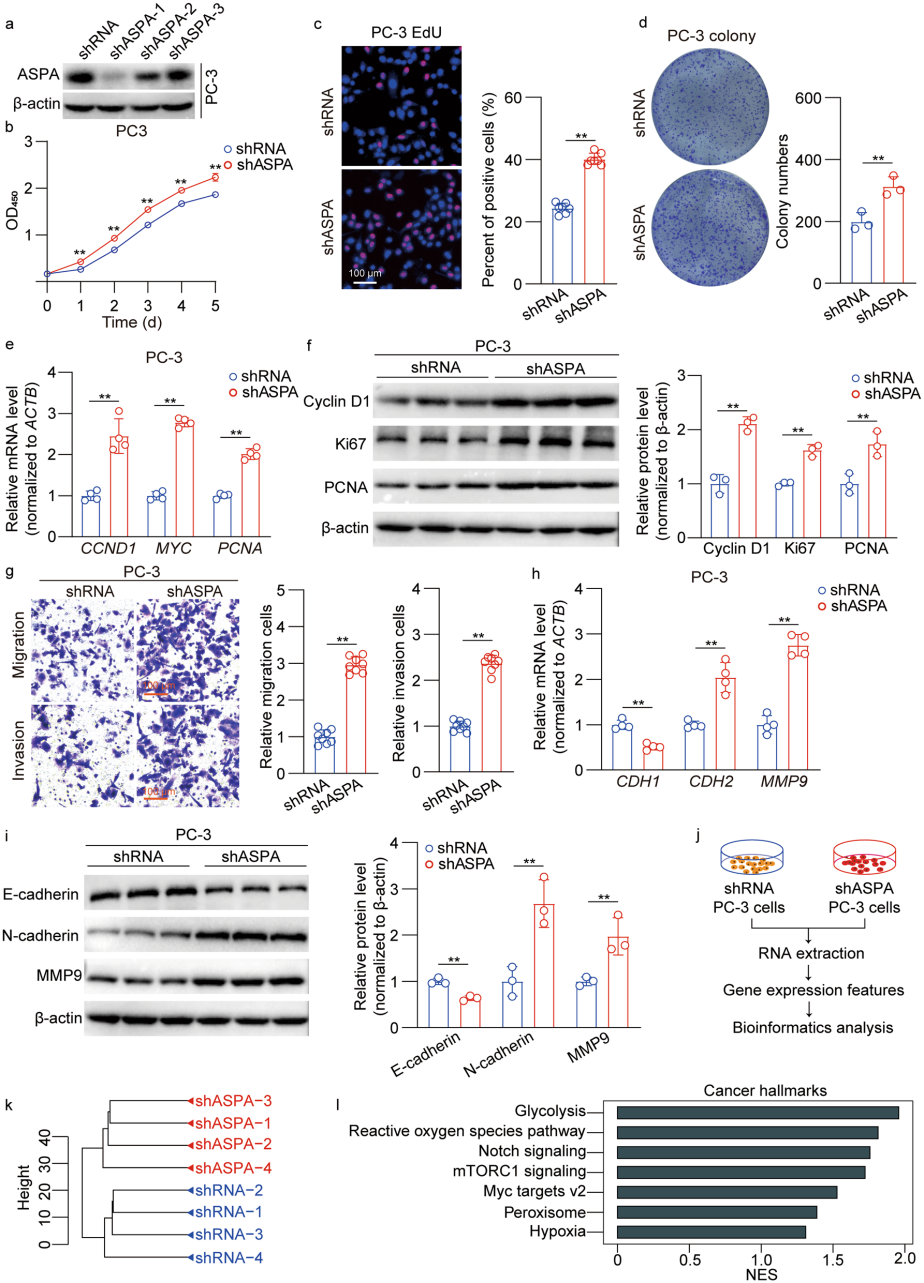
*ASPA* knockdown promotes PC-3 cell proliferation and migration in vitro. **a** Western blotting results of ASPA protein expression in PC-3 cells transfected with shRNA or shASPA. Protein expression levels were normalized to β-actin levels. **b** CCK-8 assay showed that *ASPA* knockdown promoted PC-3 cell proliferation. **c** Representative images of EdU-positive PC-3 cells transfected with shRNA or shASPA (scale bar = 100 μm). The graph on the right shows the percentage of EdU-positive nuclei. The data were obtained from 7 fields of 3 independent experiments. **d** Colony formation assay showed that *ASPA* knockdown promoted PC-3 cell colony formation ability. The graph on the right shows the colony numbers from 3 independent experiments. **e** RT-qPCR results of proliferation-related genes in PC-3 cells transfected with shRNA or shASPA. The mRNA expression levels were normalized to *ACTB* levels. **f** Western blotting results (left) and quantification results (right) for proliferation-related proteins in PC-3 cells transfected with shRNA or shASPA. Protein expression levels were normalized to β-actin levels. **g** Transwell assays showed that *ASPA* knockdown promoted PC-3 cell migration and invasion (scale bar = 100 μm). The graph on the right shows the migrating cells and the invading cells. The data were obtained from 8 fields of 3 independent experiments. **h** RT-qPCR results of epithelial–mesenchymal transition genes in PC-3 cells transfected with shRNA or shASPA. The mRNA expression was normalized to *ACTB* levels. **i** Western blotting results (left) and quantification results (right) of epithelial–mesenchymal transition proteins in PC-3 cells transfected with shRNA or shASPA. The protein expression was normalized to β-actin levels. **j** Flow chart of RNA-Seq in PC-3 cells transfected with shRNA or sh*ASPA*. **k** Cluster analysis showed the global sample distribution profiles of the shRNA group and shASPA group based on the RNA-Seq dataset in PC-3 cells. **l** GSEA results showed the significantly altered cancer hallmarks based on the RNA-Seq dataset in PC-3 cells from the shRNA group and shASPA group. The data are presented as the mean ± standard deviation (SD). ASPA aspartoacylase, CCK-8 cell counting kit 8, CCND1 cyclin D1, CDH1 cadherin 1, CDH2 cadherin 2, EdU 5-ethynyl-2′-deoxyuridine, GSEA gene set enrichment analysis, MYC v-Myc myelocytomatosis viral oncogene homolog, MMP9 matrix metallopeptidase 9, NES normalized enrichment score, OD optical density, PCNA proliferating cell nuclear antigen, RNA-Seq RNA sequencing, RT-qPCR real-time quantitative PCR, shRNA small hairpin RNA. **P < 0.01

The efficacy of *ASPA* knockdown was validated through RT-qPCR and Western blotting analyses, revealing that only shASPA-1 exhibited a significant knockdown effect and was thus selected for subsequent experimentation (Fig. 3a; Additional file 1: Fig. S3a, b). We proceeded to examine the functional consequences of *ASPA* knockdown on PCa cell proliferation and viability. In stark contrast to ASPA overexpression, *ASPA* knockdown considerably enhanced PCa cell growth and proliferation, as demonstrated by CCK-8, EdU, and colony formation assays (*P* < 0.01; Fig. 3b–d; Additional file 1: Fig. S3c–e). In agreement with these findings, RT-qPCR data indicated that *ASPA* knockdown led to elevated mRNA levels of *CCND1*, *MYC*, and *PCNA* (*P* < 0.01; Fig. 3e, Additional file 1: Fig. S3f), while Western blotting results suggested increased expression of cyclin D1, Ki67, and PCNA (*P* < 0.01; Fig. 3f, Additional file 1: Fig. S3g).

Subsequent Transwell assays revealed that *ASPA* knockdown markedly enhanced the migratory and invasive capacities of PCa cells (*P* < 0.01; Fig. 3g, Additional file 1: Fig. S3h). Additionally, *ASPA* knockdown increased the expression levels of *CDH2* and *MMP9*, while decreased *CDH1* expression (*P* < 0.01; Fig. 3h, Additional file 1: Fig. S3i). Concurrently, Western blotting analysis demonstrated that *ASPA* knockdown promoted N-cadherin and MMP9 expression, while inhibited E-cadherin expression (*P* < 0.01; Fig. 3i, Additional file 1: Fig. S3j).

RNA-Seq was performed in PC-3 cell lines transfected with the shASPA vector, with unsupervised hierarchical clustering distinctly segregating the samples into two subclusters (Fig. 3j, k). GSEA systematically disclosed that cellular signaling pathways or genes associated with glycolysis, reactive oxygen species pathway, Notch signaling, mTORC1 signaling, Myc targets v2, peroxisome, and hypoxia were significantly up-regulated by *ASPA* knockdown (*P* < 0.05 for *HK2* and *HES1*; *P* < 0.01 for *GLRX*, *PYGB*, *PGK1*, *IGFBP5*, *G6PD*, *SOD2*, *CAT*, *LFNG*, and *FZD1*; Fig. 3l, Additional file 1: Fig. S3k-m).

### ASPA suppresses the tumorigenic behavior of PCa cells in vivo

**Fig. 4:**
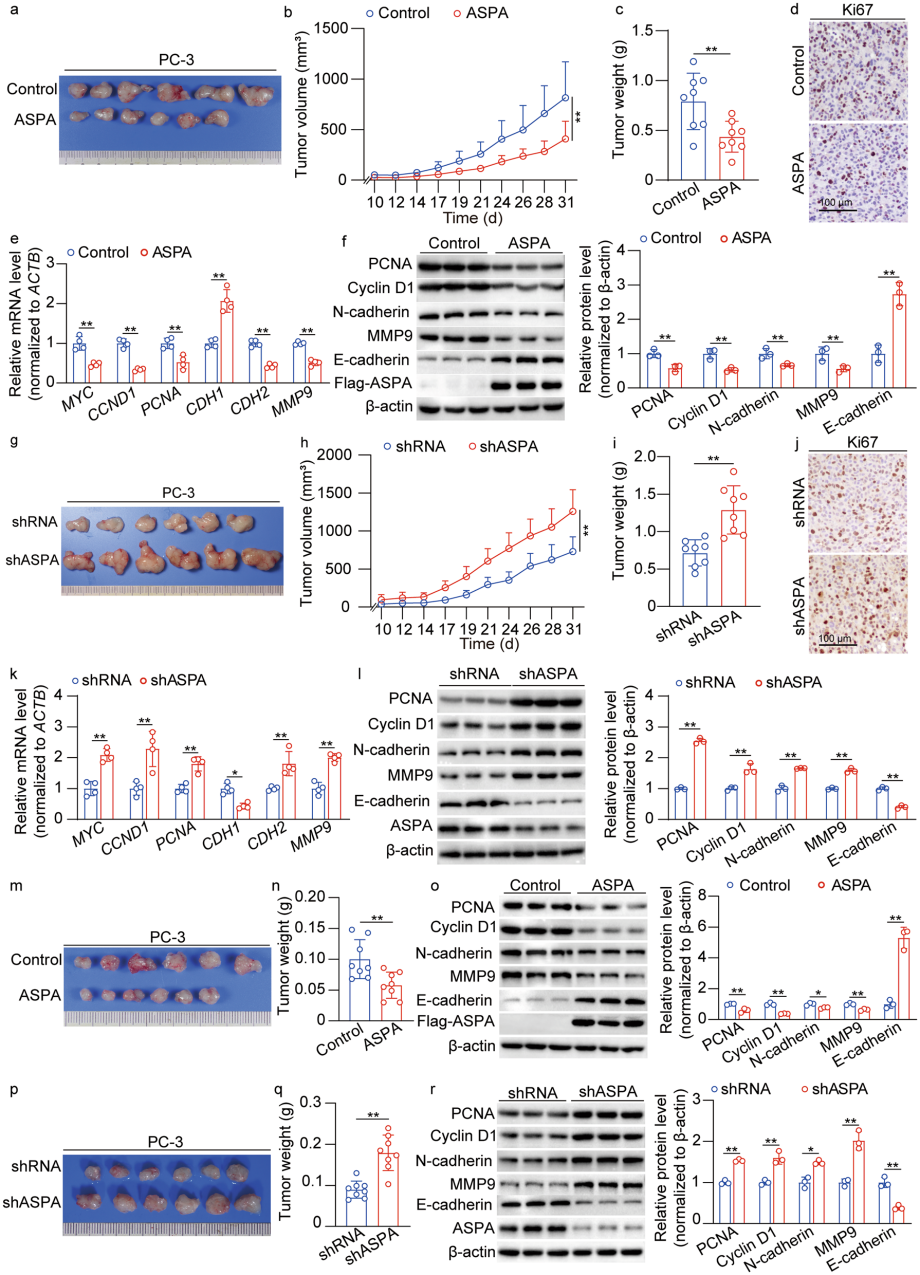
ASPA suppresses the tumorigenic behavior of PCa cells in vivo. **A** Representative images of subcutaneous xenograft tumors derived from PC-3 cells transfected with control or ASPA overexpression vector. **b** The volume of xenograft tumors in the control group and ASPA overexpression group (*n* = 8). **c** The weight of xenograft tumors in the control group and ASPA overexpression group (*n* = 8). **d** Representative images of IHC showed the expression of Ki67 in xenograft tumors in the control group and ASPA overexpression group (scale bar = 100 μm). **e** RT-qPCR results of proliferation-related genes and epithelial–mesenchymal transition genes in xenograft tumors in the control group and ASPA overexpression group. The mRNA expression levels were normalized to *ACTB* levels. **f** Western blotting results (left) and quantification (right) results for ASPA, proliferation-related proteins, and epithelial–mesenchymal transition proteins in xenograft tumors in the control group and ASPA overexpression group. Protein expression levels were normalized to β-actin levels. **g** Representative images of subcutaneous xenograft tumors derived from PC-3 cells transfected with shRNA and shASPA. **h** The volume of xenograft tumors in the shRNA group and shASPA group (*n* = 8). **i** The weight of xenograft tumors in the shRNA group and shASPA group (*n* = 8). **j** Representative images of IHC showed the expression of Ki67 in xenograft tumors in the shRNA group and shASPA group (scale bar = 100 μm). **k** RT-qPCR results of proliferation-related genes and epithelial–mesenchymal transition genes in xenograft tumors in the shRNA group and shASPA group. The mRNA expression levels were normalized to *ACTB* levels. **l** Western blotting results (left) and quantification (right) results for ASPA, proliferation-related proteins, and epithelial–mesenchymal transition proteins in xenograft tumors in the shRNA group and shASPA group. Protein expression levels were normalized to β-actin levels. **m** Representative images of orthotopic xenograft tumors derived from PC-3 cells transfected with control or ASPA overexpression vector. **n** The weight of orthotopic xenograft tumors in the control group and ASPA overexpression group (*n* = 8). **o** Western blotting results (left) and quantification (right) results for ASPA, proliferation-related proteins, and epithelial–mesenchymal transition proteins in orthotopic xenograft tumors in the control group and ASPA overexpression group. Protein expression levels were normalized to β-actin levels. **p** Representative images of orthotopic xenograft tumors derived from PC-3 cells transfected with shRNA or shASPA. **q** The weight of orthotopic xenograft tumors in the shRNA group and shASPA group (*n* = 8). **r** Western blotting results (left) and quantification (right) results for ASPA, proliferation-related proteins, and epithelial–mesenchymal transition proteins in orthotopic xenograft tumors in the shRNA group and shASPA group. Protein expression levels were normalized to β-actin levels. The data are presented as the mean ± standard deviation (SD). ASPA aspartoacylase, CCND1 cyclin D1, CDH1 Cadherin 1, CDH2 Cadherin 2, IHC immunohistochemistry, PCa prostate cancer, MYC v-Myc myelocytomatosis viral oncogene homolog, MMP9 matrix metallopeptidase 9, PCNA proliferating cell nuclear antigen, RT-qPCR real-time quantitative PCR, shRNA small hairpin RNA. *P < 0.05, **P < 0.01

In tumor-bearing murine models, overexpression of ASPA significantly reduced tumor volumes and weights (*P* < 0.01; Fig. 4a–c). IHC staining indicated a markedly lower number of Ki67-positive cells in the ASPA overexpression group compared to the control (Fig. 4d). Furthermore, the RT-qPCR results revealed that ASPA overexpression down-regulated the mRNA levels of *MYC*, *CCND1*, *PCNA*, *CDH2*, and *MMP9*, while up-regulated *CDH1* expression (*P* < 0.01; Fig. 4e). Concurrently, Western blotting analyses demonstrated that ASPA overexpression attenuated the expression of PCNA, cyclin D1, N-cadherin, and MMP9, but enhanced E-cadherin expression (*P* < 0.01; Fig. 4f).

Conversely, *ASPA* knockdown led to increased tumor volumes and weights (*P* < 0.01; Fig. 4g–i), with IHC staining exhibiting a significantly higher number of Ki67-positive cells in the *ASPA* knockdown group compared to the control (Fig. 4j). Additionally, RT-qPCR results indicated that *ASPA* knockdown up-regulated the mRNA levels of *MYC*, *CCND1*, *PCNA*, *CDH2*, and *MMP9* (*P* < 0.01), while down-regulated *CDH1* (*P* < 0.05) expression (Fig. 4k). Simultaneously, Western blotting analyses revealed that *ASPA* knockdown increased the expression of PCNA, cyclin D1, N-cadherin, and MMP9, but decreased E-cadherin expression (*P* < 0.01; Fig. 4l).

Moreover, orthotopic xenograft models demonstrated that ASPA overexpression led to a decrease in tumor weight (*P* < 0.01; Fig. 4m, n). Western blotting analyses further corroborated that ASPA overexpression suppressed the expression of PCNA, cyclin D1, N-cadherin, and MMP9 (*P* < 0.01), while promoting E-cadherin expression (*P* < 0.01; Fig. 4o). In contrast, *ASPA* knockdown resulted in increased tumor weights and up-regulated expression of PCNA, cyclin D1, N-cadherin, and MMP9 (*P* < 0.05 or *P* < 0.01), concomitant with the inhibition of E-cadherin expression (*P* < 0.01; Fig. 4p–r).

### ASPA negatively regulates JNK1/2-C-Jun signaling

**Fig. 5:**
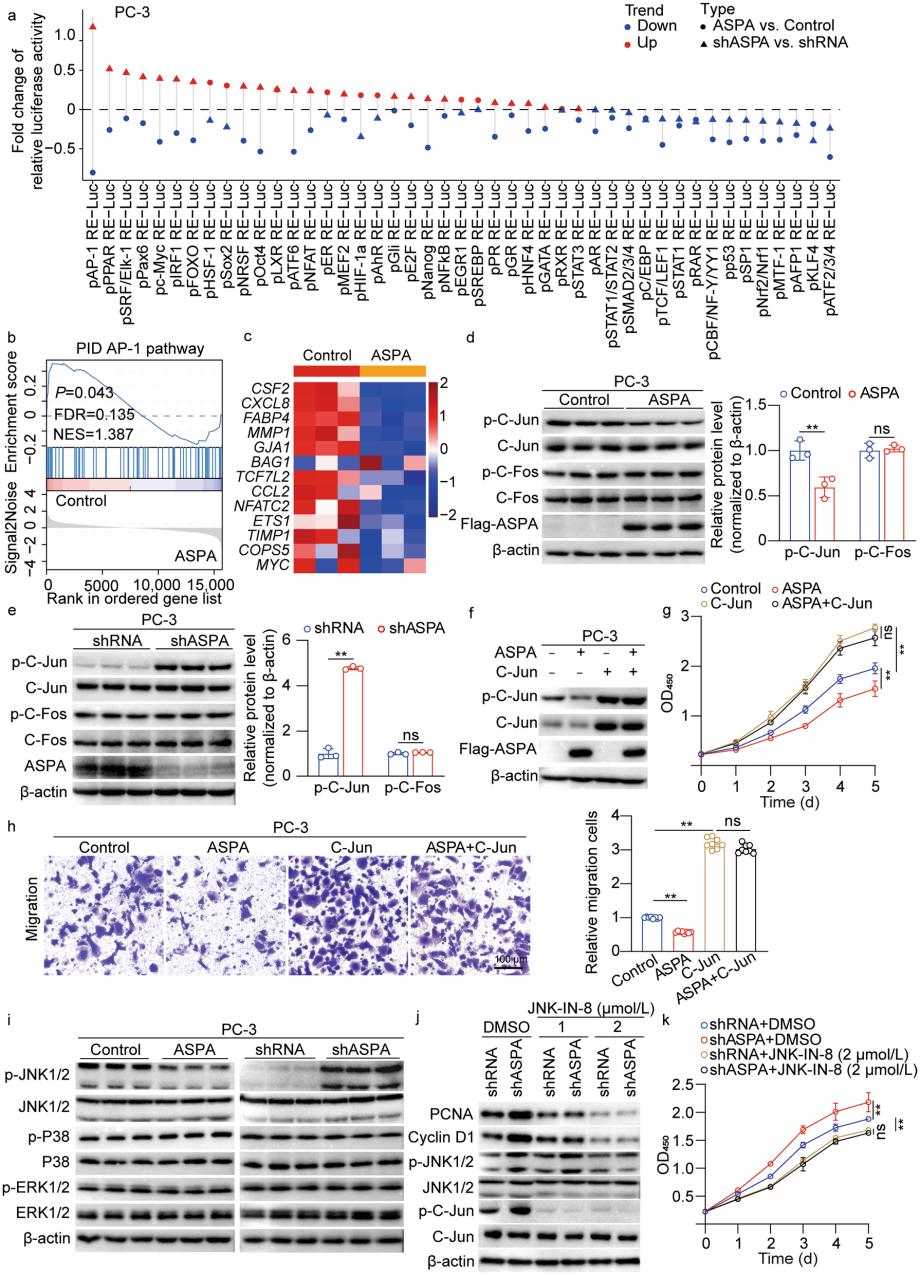
ASPA negatively regulates JNK1/2-C-Jun activity. **a** Luciferase activity of 45 pathways affected by ASPA overexpression or knockdown. Blue and red indicate down- and up-regulated activity, respectively. Dot and triangle indicate ASPA versus control and shASPA versus shRNA, respectively. **b** Enrichment of the AP-1 pathway in the control group and ASPA overexpression group and analysis by GSEA based on RNA-Seq dataset in PC-3 cells. **c** Heatmap showed the significantly altered genes related to the AP-1 pathway based on the RNA-Seq dataset in PC-3 cells transfected with the control or ASPA overexpression vector. **d** Western blotting results (left) and quantification (right) results for ASPA and phosphorylation of AP-1 (C-Jun and C-Fos) in PC-3 cells transfected with control or ASPA overexpression vectors. Protein expression levels were normalized to β-actin levels. **e** Western blotting results (left) and quantification (right) results for ASPA and phosphorylation of AP-1 (C-Jun and C-Fos) in PC-3 cells transfected with shRNA or shASPA. Protein expression levels were normalized to β-actin levels. **f** Western blotting results for ASPA and phosphorylation of C-Jun in PC-3 cells transfected with control, ASPA overexpression vector, or C-Jun overexpression vector. Protein expression levels were normalized to β-actin levels. **g** Cell proliferation ability of PC-3 cells cotransfected with ASPA overexpression vector and/or C-Jun overexpression vector was assessed using a CCK-8 assay. **h** The cell migration ability of PC-3 cells cotransfected with ASPA overexpression vector and/or C-Jun overexpression vector was assessed using a Transwell assay. The graph on the right shows the migration of cells in the treatment group relative to the control group. The data were obtained from 8 fields of 3 independent experiments (scale bar = 100 μm). **i** Western blotting results of JNK1/2, P38, and ERK1/2 phosphorylation in PC-3 cells transfected with control or ASPA overexpression vector and transfected with shRNA or shASPA. Protein expression levels were normalized to β-actin levels. **j** Western blotting analysis showed the expression of PCNA and cyclin D1 and the activity of JNK1/2 and C-Jun in PC-3 cells transfected with shASPA and/or treated with the JNK inhibitor JNK-IN-8. **k** The proliferation ability of PC-3 cells transfected with shASPA and/or treated with JNK-IN-8 was assessed using a CCK-8 assay. The data are presented as the mean ± standard deviation (SD). ASPA aspartoacylase, AP-1 activator protein-1, CCK-8 cell counting kit 8, C-Jun v-Jun avian sarcoma virus 17 oncogene homolog, C-Fos v-Fos FBJ murine osteosarcoma viral oncogene homolog, DMSO dimethyl sulfoxide, ERK extracellular regulated protein kinases, GSEA gene set enrichment analysis, FDR false discovery rate, JNK c-Jun N-terminal kinase, NES normalized enrichment score, OD optical density, PCNA proliferating cell nuclear antigen, PID Pathway Interaction Database, RT-qPCR real-time quantitative PCR, shRNA small hairpin RNA. **P < 0.01, *ns* not significant

Luciferase assays demonstrated a reduction in AP-1 reporter luciferase activity upon ASPA overexpression and an elevation following *ASPA* knockdown, suggesting a potential regulatory role for ASPA on AP-1 activity (Fig. 5a). The RNA-Seq data further substantiated these findings, revealing suppression of the AP-1 pathway with ASPA overexpression and activation upon *ASPA* knockdown (*P* = 0.043 for ASPA overexpression; *P* = 0.034 for *ASPA* knockdown; Fig. 5b, c; Additional file 1: Fig. S4a, b), thus indicating a negative regulatory effect of ASPA on AP-1 pathway activation. To gain deeper insight into the impact of ASPA on the activation of C-Jun and C-Fos, which have been identified as the primary subunits of AP-1, we conducted Western blotting analysis. Our results demonstrated that C-Jun phosphorylation was inhibited by ASPA overexpression (*P* < 0.01), while p–C-Fos levels remained unaltered (Fig. 5d). Conversely, *ASPA* knockdown resulted in enhanced C-Jun activation (*P* < 0.01; Fig. 5e). To determine if C-Jun activation is essential for ASPA-mediated inhibition of PCa cell proliferation and migration, we conducted further experiments. Western blotting analysis revealed that C-Jun activity was restored through C-Jun overexpression in ASPA-overexpressing PC-3 cells (Fig. 5f). Strikingly, C-Jun overexpression completely reversed the inhibitory effect of ASPA overexpression on PCa cell proliferation and migration (*P* < 0.01; Fig. 5g, h; Additional file 1: Fig. S4c).

Our Western blotting analyses revealed that ASPA overexpression led to the inhibition of JNK1/2 activation (*P* < 0.01; Fig. 5i, Additional file 1: Fig. S4d). However, ASPA overexpression did not appear to influence the activation of P38 and ERK1/2. Moreover, *ASPA* knockdown resulted in increased JNK1/2 activation without affecting P38 and ERK1/2 activation. To further explore whether JNK1/2 activation mediated the inhibitory effects of ASPA on C-Jun activation and PCa cell proliferation and migration, we utilized JNK inhibitor JNK-IN-8 to block JNK1/2 activation and examined the consequences of *ASPA* knockdown on cell proliferation and migration. Our results demonstrated that JNK-IN-8 counteracted the reduction in phosphorylation levels of JNK and C-Jun, induced by *ASPA* knockdown, in a dose-dependent manner, as evidenced by Western blotting (Fig. 5j). In parallel, JNK1/2 inhibition abrogated the growth advantage and enhanced migration effect prompted by *ASPA* knockdown in PCa cells (*P* < 0.01; Fig. 5k; Additional file 1: Fig. S4e, f).

### ASPA directly interacts with LYN and ASPA expression is negatively correlated with LYN phosphorylation

**Fig. 6:**
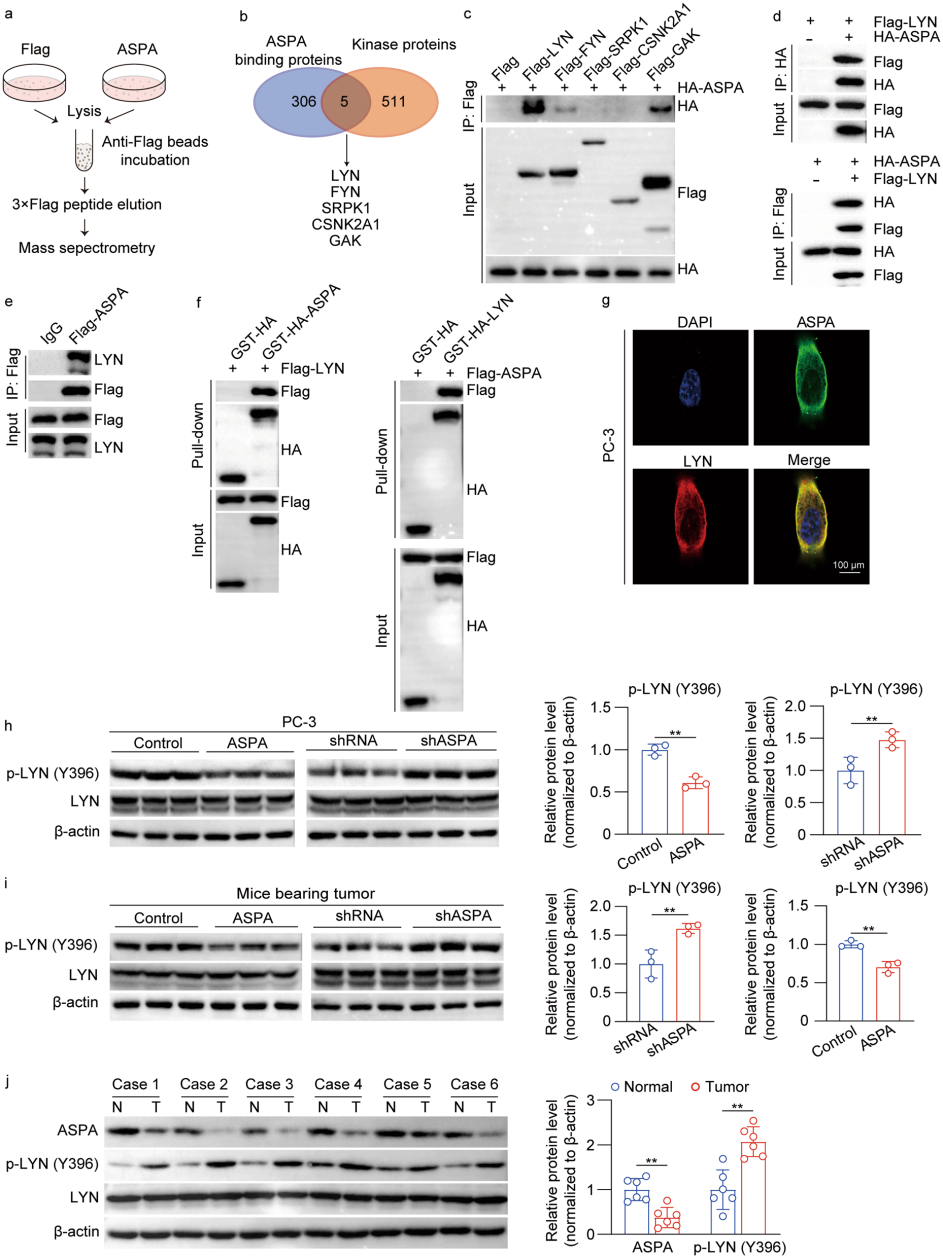
ASPA directly interacts with LYN and suppresses its phosphorylation. **a** Flow chart of the mass spectrometry analysis of ASPA-overexpressing PC-3 cells and control cells. **b** Five overlapping proteins were identified between ASPA binding proteins (306) and kinase proteins (511) in HEK293T cells. **c** Co-IP analysis of the interaction between ASPA and the indicated Flag-tagged five kinase proteins in PC-3 cells. **d** Exogenous IP assays were performed to evaluate the binding of ASPA and LYN in PC-3 cells transfected with Flag-LYN or Flag-ASPA. **e** Endogenous IP assays were performed in HEK293T cells transfected with Flag-LYN or HA-ASPA. **f** GST pull-down assays showed the direct binding of ASPA to LYN using GST-HA-ASPA and Flag-LYN (left) or using GST-HA-LYN and Flag-ASPA (right). **g** The colocalization of ASPA and LYN in PC-3 cells was determined by immunofluorescence (scale bar = 100 μm). **h** Western blotting results (left) and quantification results (right) of the phosphorylation of LYN in PC-3 cells transfected with control and ASPA overexpression vector or shRNA and shASPA. Protein expression levels were normalized to β-actin levels. **i** Western blotting results (left) and quantification results (right) of the phosphorylation of LYN in xenograft tumors derived from PC-3 cells transfected with control or ASPA overexpression vector or shRNA and shASPA. Protein expression levels were normalized to β-actin levels. **j** Western blotting results (left) and quantification results (right) of ASPA and the phosphorylation of LYN in 6 PCa samples with paired normal tissues. Protein expression levels were normalized to β-actin levels. The data are presented as the mean ± standard deviation (SD). ASPA aspartoacylase, Co-IP coimmunoprecipitation, CSNK2A1 casein kinase 2 alpha 1, DAPI 4′,6-Diamidino-2-phenylindole dihydrochloride, FYN tyrosine-protein kinase Fyn, GAK cyclin G associated kinase, GST glutathione S-transferase, HEK-293T human embryonic kidney cell 293T, IP immunoprecipitation, LYN Lck/Yes-related novel protein tyrosine kinase, PCa prostate cancer, shRNA small hairpin RNA, SRPK1 serine/arginine-rich protein-specific kinase 1. **P < 0.01

The mass spectrometry analyses disclosed 306 proteins that potentially interacted with ASPA (Fig. 6a). In vitro evidence demonstrated that ASPA overexpression influenced the activation of JNK1/2 and C-Jun through phosphorylation modification. Consequently, we aimed to delineate the kinase proteins that may interact with ASPA. Among the 306 interacting proteins, a mere 5 kinase proteins were identified (LYN, FYN, SRPK1, CSNK2A1, and GAK) and selected for evaluation of their interaction with ASPA using Co-IP analyses (Fig. 6b). The findings indicated that LYN displayed the highest affinity for ASPA binding in comparison to other kinase proteins (Fig. 6c). The ASPA-LYN interaction was substantiated by reciprocal exogenous Co-IP assays (Fig. 6d) and endogenous IP assays in the PC-3 cell lines (Fig. 6e). This observation was further corroborated by GST pull-down assays employing purified recombinant proteins, which revealed a direct binding between ASPA and LYN (Fig. 6f). Furthermore, the colocalization of ASPA and LYN in PC-3 cells was visualized via immunofluorescence assays, signifying the interaction between ASPA and LYN in prostates (Fig. 6g). The influence of ASPA on LYN activity was subsequently investigated. Overexpression of ASPA suppressed LYN phosphorylation, while its knockdown augmented LYN activity in PC-3 cells, as evidenced by Western blotting analysis (*P* < 0.01; Fig. 6h). The modulation of LYN activity by ASPA in mice bearing PC-3 cell-derived tumors was examined, revealing that ASPA overexpression inhibited LYN (Y396) phosphorylation, whereas *ASPA* knockdown enhanced LYN activity in these mice (*P* < 0.01; Fig. 6i). Moreover, LYN (Y396) activation was observed in PCa patients exhibiting ASPA downregulation (*P* < 0.01; Fig. 6j).

### LYN inhibition is necessary for the protective roles of ASPA in PCa

**Fig. 7:**
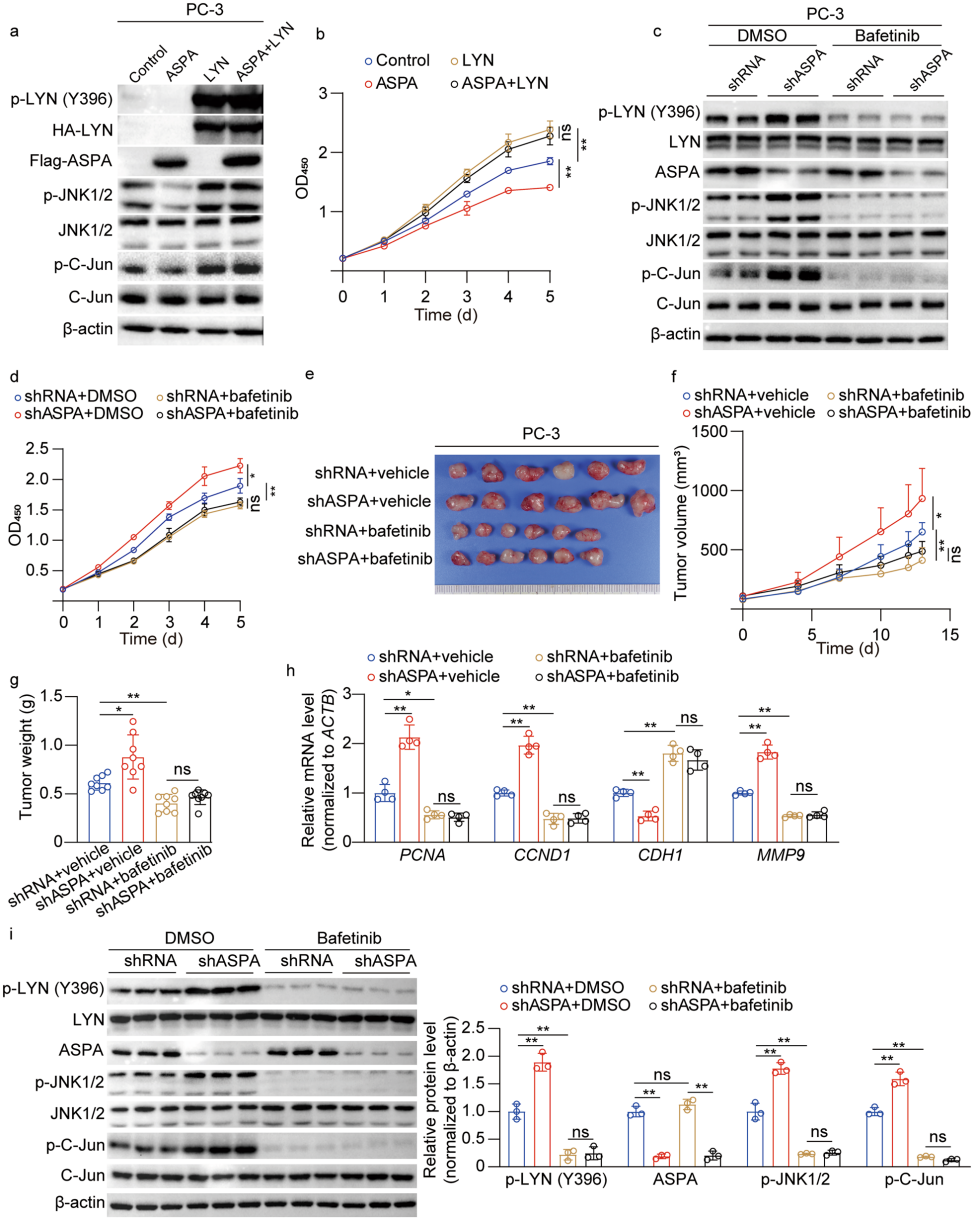
LYN mediates the function of ASPA in PCa cells. **a** Western blotting assay showed ASPA expression and the activity of LYN, JNK1/2, and C-Jun in PC-3 cells cotransfected with ASPA overexpression vector and/or LYN overexpression vector. **b** The cell proliferation ability of PC-3 cells cotransfected with ASPA overexpression vector and/or LYN overexpression vector was assessed using CCK-8 assay. **c** Western blotting assay showed ASPA expression and the activity of LYN, JNK1/2, and C-Jun in PC-3 cells transfected with shASPA and/or treated with the LYN inhibitor bafetinib. **d** The proliferation ability of PC-3 cells transfected with shASPA and/or treated with bafetinib was assessed using a CCK-8 assay. **e** Representative images of subcutaneous xenograft tumors derived from PC-3 cells transfected with shASPA and/or treated with bafetinib. **f** The volume of xenograft tumors derived from PC-3 cells transfected with shASPA and/or treated with bafetinib (*n* = 8). **g** The weight of xenograft tumors derived from PC-3 cells transfected with shASPA and/or treated with bafetinib (*n* = 8). **h** RT-qPCR results of proliferation-related genes and epithelial–mesenchymal transition genes in xenograft tumors derived from PC-3 cells transfected with shASPA and/or treated with bafetinib. The mRNA expression levels were normalized to *ACTB* levels. **i** Western blotting analysis (left) and quantification (right) of ASPA and the phosphorylation of LYN, JNK1/2, and C-Jun in subcutaneous xenograft tumors derived from PC-3 cells transfected with shASPA and/or treated with bafetinib. Protein expression levels were normalized to β-actin levels. The data are presented as the mean ± standard deviation (SD). ASPA aspartoacylase, CCND1 cyclin D1, CDH1 Cadherin 1, CCK-8 cell counting kit 8, C-Jun v-Jun avian sarcoma virus 17 oncogene homolog, DMSO dimethyl sulfoxide, JNK c-Jun N-terminal kinase, LYN Lck/Yes-related novel protein tyrosine kinase, OD optical density, RT-qPCR real-time quantitative PCR, PCNA proliferating cell nuclear antigen, shRNA small hairpin RNA. *P < 0.05, **P < 0.01, ns not significant

In light of the observed inhibition of LYN activation by ASPA, we sought to investigate the necessity of LYN inhibition for ASPA-mediated protective roles in PCa by overexpressing LYN in ASPA-overexpressing cells. Western blotting analysis demonstrated that both LYN (Y396) phosphorylation and downstream JNK1/2-C-Jun levels were restored following LYN overexpression in ASPA-overexpressing cells (Fig. 7a). Furthermore, the inhibitory effects on cell proliferation and migration attributed to ASPA overexpression were abrogated by LYN overexpression (*P* < 0.01; Fig. 7b; Additional file 1: Fig. S5a, b). Given the heightened LYN activation in *ASPA* knockdown cells, we employed bafetinib, a LYN inhibitor, to evaluate LYN’s influence on PC-3 cell lines. Western blotting analysis revealed a reduction in phosphorylation levels of LYN (Y396), JNK1/2, and C-Jun upon bafetinib treatment in shASPA cells (Fig. 7c). Notably, bafetinib also abrogated the growth- and migration-promoting effects associated with *ASPA* knockdown (*P* < 0.01; Fig. 7d; Additional file 1: Fig. S5c, d). The critical role of LYN in PCa progression in vivo was further substantiated, and bafetinib was shown to counteract the promoting effects of *ASPA* knockdown in murine models (Fig. 7e–g). RT-qPCR, immunohistochemical staining, and Western blotting analyses collectively demonstrated that the upregulation of *PCNA*, *CCND1*, *CDH1*, and *MMP9* mRNA levels, the increased proportion of Ki67-positive cells, and the activation of LYN (Y396), JNK1/2, and C-Jun induced by *ASPA* knockdown in subcutaneous xenograft tumors derived from PC-3 cells were all counteracted by bafetinib treatment (Fig. 7h, i; Additional file 1: Fig. S5e).

### ASPA regulates LYN activity in an enzyme-independent manner

**Fig. 8:**
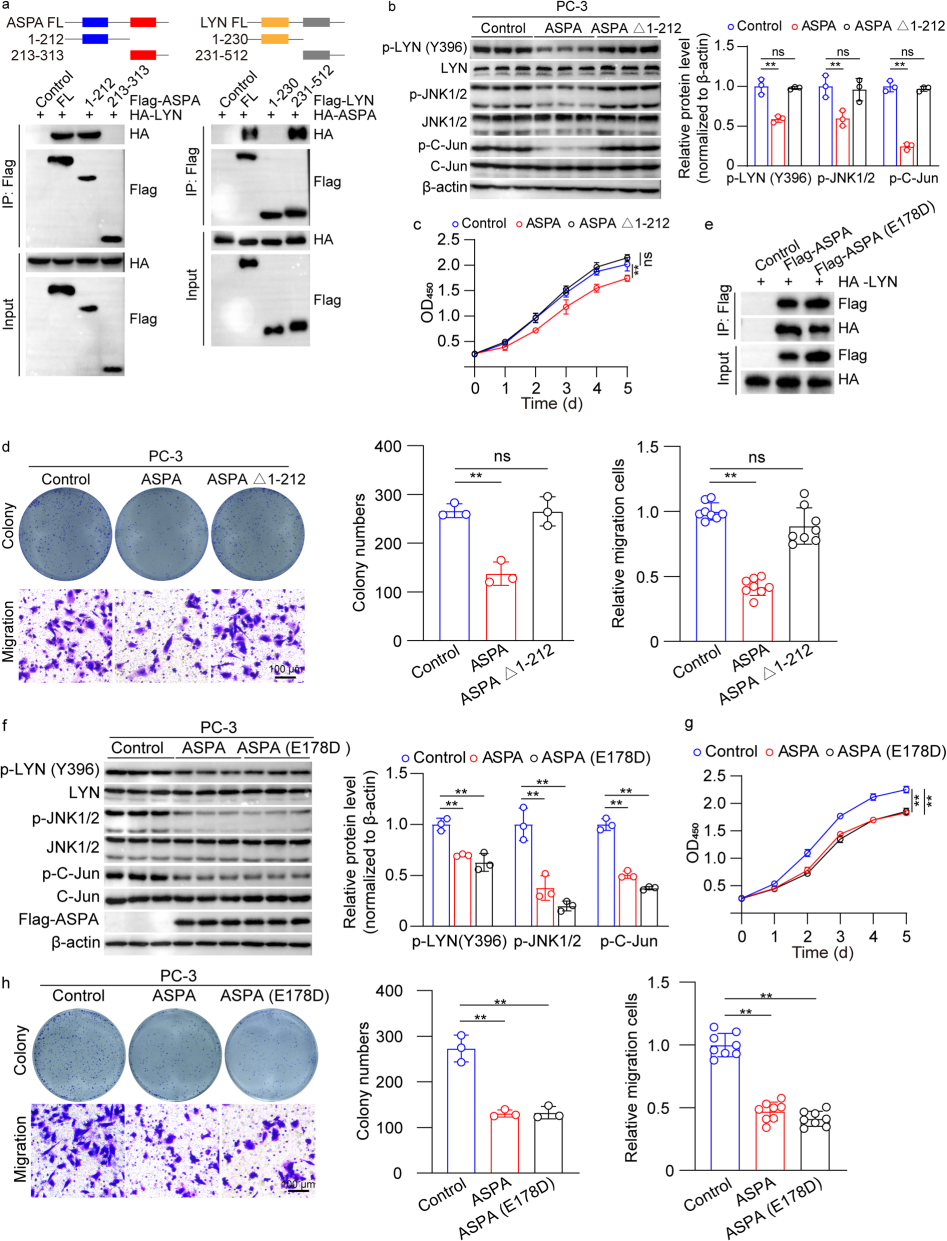
ASPA interacts with the N-terminus of LYN and regulates its activity in an enzyme-independent manner. **a** The interaction domain(s) between ASPA (left) and LYN (right) were determined by IP assays in HEK293T cells using full-length and truncated LYN or ASPA expression constructs. **b** Western blotting analysis (top) and quantification (bottom) of the phosphorylation of LYN, JNK1/2, and C-Jun in PC-3 cells transfected with the Flag-ASPA and Flag-ASPA Δ1-212 (deletion of amino acids 1-222) vectors. Protein expression levels were normalized to β-actin levels. **c** The proliferation ability of PC-3 cells transfected with Flag-ASPA and Flag-ASPA Δ1-212 vectors was assessed using a CCK-8 assay. **d** Cell colony formation ability (top) and migration ability (bottom) of PC-3 cells transfected with Flag-ASPA and Flag-ASPA Δ1-212 vectors were assessed using colony formation and Transwell assays. The graph on the right shows the colony numbers (left) from 3 independent experiments and the number of migrated cells (right) obtained from 8 fields of 3 independent experiments (scale bar = 100 μm). **e** Co-IP assays showed that the interaction between ASPA and LYN was not affected by the E178D mutation in HEK293T cells cotransfected with HA-LYN, Flag-ASPA and Flag-ASPA E178D vectors. **f** Western blotting analysis (left) and quantification (right) of ASPA and the phosphorylation of LYN, JNK1/2, and C-Jun in PC-3 cells transfected with the Flag-ASPA and Flag-ASPA E178D vectors. Protein expression levels were normalized to β-actin levels. **g** The proliferation ability of PC-3 cells transfected with Flag-ASPA and Flag-ASPA E178D vectors was assessed using a CCK-8 assay. **h** Cell colony formation ability (top) and migration ability (bottom) of PC-3 cells transfected with Flag-ASPA and Flag-ASPA E178D vectors were assessed using colony formation and Transwell assays. The graph below shows the colony numbers (left) from 3 independent experiments and the number of migrated cells (right) obtained from 8 fields of 3 independent experiments (scale bar = 100 μm). **i** The mechanism of ASPA in PCa. The data are presented as the mean ± standard deviation (SD). ASPA aspartoacylase, CCK-8 cell counting kit 8, C-Jun v-Jun avian sarcoma virus 17 oncogene homolog, Co-IP coimmunoprecipitation, HEK-293 T human embryonic kidney cell 293 T, JNK c-Jun N-terminal kinase, LYN Lck/Yes-related novel protein tyrosine kinase, OD optical density, PCa prostate cancer. **P < 0.01, ns not significant

We further substantiated that the kinase domain (N-terminal residues 231-512) of LYN, rather than the Src homologs (SH2 and SH3), was accountable for the interaction with ASPA (Fig. 8a). Subsequently, we sought to discern the specific ASPA domain required for LYN interaction. Protein domain analyses revealed a direct interaction between N-terminal residues 1-212 of ASPA and C-terminal residues 231-512 of LYN (Fig. 8a). To assess the necessity of N-terminal residues 1-212 of ASPA in PCa cells, we transfected the ASPA vector and constructed an ASPA vector with N-terminal residues 1-212 deletion (ASPA Δ1-212). Western blotting results demonstrated that ASPA Δ1-212 did not influence LYN (Y396), JNK1/2, or C-Jun phosphorylation in PC-3 cell lines (*P* < 0.01; Fig. 8b). Furthermore, ASPA Δ1-212 did not impact cell proliferation and migration in PC-3 cell lines (*P* < 0.01; Fig. 8c, d), signifying that N-terminal residues 1–212 of ASPA were crucial for ASPA’s inhibitory effects.

To examine whether the protective role of ASPA in PCa relied on its classical function, we generated an E178D mutation of ASPA (Glu178Asp), which substantially contributes to ASPA’s enzymatic activity. Co-IP assays indicated that the interaction between ASPA and LYN remained unaffected by the E178D mutation (Fig. 8e). We then transfected cells with vectors containing wild-type ASPA and ASPA with the E178D mutation to assess the necessity of ASPA’s enzymatic activity in PCa cells. Western blotting results uncovered that the E178D mutation of ASPA inhibited LYN (Y396), JNK1/2, and C-Jun phosphorylation levels in PC-3 cell lines (*P* < 0.01; Fig. 8f). Moreover, the E178D mutation of ASPA also inhibited cell proliferation and migration in PC-3 cell lines (*P* < 0.01; Fig. 8g, h).

## Discussion

In our investigation, we have demonstrated that ASPA functioned as a tumor suppressor in PCa. The expression levels of *ASPA* mRNA were found to be significantly reduced in PCa tissues compared to adjacent non-cancerous tissues, and these levels were correlated with malignant PCa phenotypes, such as the T and N stages as well as the Gleason score. This evidence suggests a role for ASPA in the progression of PCa. Moreover, *PTEN* and *SPOP* have been previously identified as suppressors of PCa [25, 26]. We discovered a positive correlation between the expression of *ASPA*, *PTEN*, and *SPOP*, further supporting the potential inhibitory function of ASPA in PCa. Both in vitro and in vivo experiments revealed that overexpression of ASPA attenuated the progression of PCa, whereas *ASPA* knockdown significantly enhanced the malignant phenotypes of PCa. Furthermore, our research demonstrated that ASPA directly interacts with the LYN kinase and impedes the phosphorylation of LYN (Y396), JNK1/2, and C-Jun, thereby inhibiting the progression of PCa. We observed that ASPA expression was down-regulated in a substantial number of PCa patients, while LYN activity was elevated in these individuals. Consequently, the inhibition of LYN presents a promising therapeutic strategy for PCa patients with down-regulated ASPA expression and heightened LYN activity. Collectively, our study offers potential therapeutic targets and novel approaches for the treatment of PCa, underscoring the significance of understanding the underlying molecular mechanisms involved in its progression.

The effective clinical management of PCa, particularly metastatic cancer, poses a significant challenge due to the limited knowledge of its molecular mechanisms underlying cancer progression. Consequently, understanding the precise molecular mechanisms behind PCa progression is crucial for developing enhanced management strategies for PCa patients. Over recent decades, numerous potential molecular targets for PCa therapy have been identified [27–34]. Accumulating evidence implicates LYN, a member of the Src family of tyrosine kinases, as a target gene for PCa, given its role in regulating cell proliferation, migration, and invasion [35–40]. Tatarov et al. [41] reported that hormone-refractory PCa patients with elevated Src family kinase activity experienced significantly reduced overall survival. Furthermore, high expression of Src family kinases correlated with the presence of distant metastases. Park et al. [39] discovered that *LYN* knockdown substantially diminished PCa cell proliferation in vitro. However, the READY trial [42], a randomized, double-blind phase III trial involving 1522 eligible patients from 186 centers across 25 countries, revealed that dasatinib, a nonspecific tyrosine kinase inhibitor with 40-fold higher selectivity for Src than for LYN [43], did not impact overall survival in patients with metastatic CRPC. This finding suggests that targeting LYN may prove more effective than targeting Src.

Conversely, complete deletion or blockade of LYN promotes the inflammatory cytokine response, resulting in heightened susceptibility to *Pseudomonas aeruginosa* infection [44]. As such, a promising strategy for treating PCa may involve inhibiting LYN hyperactivation through dephosphorylation without altering LYN’s physiological activity. In this study, we report for the first time that ASPA binds to LYN and suppresses JNK1/2-AP-1-C-Jun downstream activity. Our findings indicate that the regulatory effect of ASPA on PCa is independent of its enzymatic activity. Rather, we determined that ASPA modulates PCa progression by directly interacting with LYN, a critical kinase protein involved in protein phosphorylation, and by inhibiting LYN’s phosphorylation. The nonenzymatic activity of ASPA has not been previously documented. Consequently, we postulate that inducing ASPA expression, an inhibitor of LYN activation, could represent a potential approach for regulating LYN hyperactivation during PCa progression.

We identified that ASPA is down-regulated in PCa tissues relative to adjacent tissues, as determined by transcriptional analysis of a public dataset from the TCGA and our own RNA-Seq dataset. ASPA is an enzyme responsible for catalyzing the conversion of *N*-acetyl-l-aspartic acid to aspartate and acetate. Mutations in this gene result in the accumulation of *N*-acetyl-l-aspartic acid, leading to Canavan disease [14, 45]. Only one study has investigated the ASPA level and prognosis of cancer in neuroblastoma patients [10]. To the best of our knowledge, our research is the first to establish that ASPA functions as a suppressor of PCa.

Furthermore, we explored whether ASPA binds to LYN and attenuates the activity of its downstream targets. Notably, we discovered that amino acids 1-212 of the N-terminus of ASPA directly interact with amino acids 231-512 of the C-terminus of LYN, and that this domain mediates the inhibitory effects of ASPA on JNK1/2-C-Jun signaling cascades. To ascertain whether the protective role of ASPA in PCa was dependent on its classical enzyme function, we constructed the E178D mutation of ASPA (Glu178Asp), which significantly contributes to ASPA’s enzymatic activity [46]. Our findings revealed that the enzymatic activity of ASPA does not play a role in the interaction between ASPA and LYN, nor does it contribute to LYN activation. Most importantly, we observed that LYN was highly activated in PCa patients with low ASPA expression. We speculated that LYN inhibition might be valuable in treating PCa patients with low ASPA expression.

While the proposed study aims to provide valuable insights into the mechanistic role of ASPA in PCa progression and its potential as a therapeutic target, there are several limitations that should be considered. Firstly, although human PCa cell lines can offer valuable information, they may not fully recapitulate the complex tumor microenvironment present in vivo. Consequently, the results obtained from in vitro experiments may not always translate to in vivo settings. Secondly, although the use of two mice models may help validate the protective effect of ASPA against PCa progression, these models may not entirely represent human PCa biology. Differences in species and their response to interventions can lead to discrepancies in the results when translating findings to human subjects. Thirdly, the development of pharmacological agents targeting LYN and their testing in preclinical models and early-phase clinical trials are subject to multiple challenges. These may include difficulties in drug design, optimization of drug delivery, potential off-target effects, and unforeseen toxicities, which could hinder the successful translation of LYN-targeting therapies into clinical practice. Lastly, the study focuses on mechanistic evidence supporting the association between ASPA and PCa progression and the potential therapeutic target of ASPA for PCa management, which may not cover all aspects of PCa biology or other potential therapeutic targets. A comprehensive understanding of the disease requires the investigation of multiple molecular pathways and targets. Therefore, while the proposed study has the potential to provide valuable insights into the role of ASPA in PCa progression and its therapeutic potential, the aforementioned limitations must be considered when interpreting the results and designing future research.

## Conclusions

**Fig. 9:**
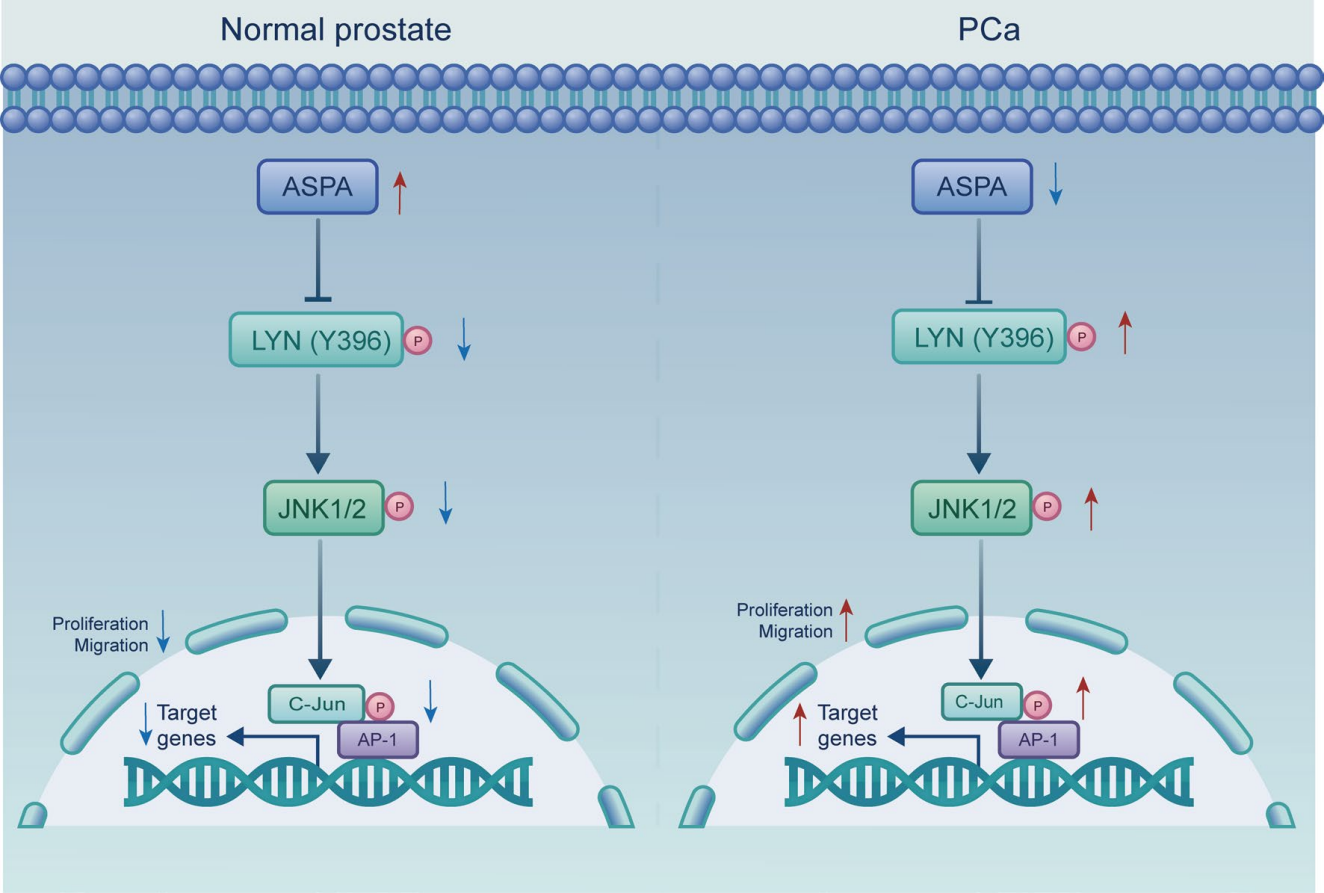
The mechanism of ASPA in PCa. ASPA aspartoacylase, AP-1 Activator protein-1, C-Jun v-Jun avian sarcoma virus 17 oncogene homolog, JNK c-Jun N-terminal kinase, LYN Lck/Yes-related novel protein tyrosine kinase, PCa prostate cancer

The objective of this investigation is to elucidate the mechanistic evidence substantiating the correlation between ASPA and PCa progression, as well as to assess the potential of ASPA as a viable therapeutic target for the management of PCa. The findings of the current study delineate a previously uncharacterized ASPA-LYN-JNK1/2-AP-1/c-Jun signaling pathway implicated in the progression of PCa (Fig. 9). Significantly, the data demonstrate that ASPA exerts an inhibitory effect on PCa progression not solely through its inherent enzymatic function, but also by directly interacting with LYN and impeding its phosphorylation, which represents a heretofore unidentified mechanism attributable to ASPA.

## Supplementary Information

**Additional file 1: Table S1.** Characteristics of patients providing samples for RNA-Seq. **Table S2.** Characteristics of patients providing samples for Western blotting. **Table S3.** Characteristics of patients providing samples for IHC. **Table S4.** Characteristics of patients providing samples for RT-qPCR. **Fig. S1.** ASPA is down-regulated in PCa patients. **Fig. S2.** ASPA overexpression inhibits DU145 cell proliferation and migration in vitro. **Fig. S3.** *ASPA* knockdown promotes DU145 cell proliferation and migration in vitro. **Fig. S4.** ASPA negatively regulates JNK1/2-C-Jun activity in PC-3 cells. **Fig. S5.** LYN mediates the function of ASPA in PC-3 cells.

## Data Availability

The data that support the findings of this study are available from the corresponding author on reasonable request.

## Abbreviations

ACY1: Aminoacylase 1
ANOVA: Analysis of variance
ASPA: Aspartoacylase
AP-1: Activator protein-1
BCA: Bicinchoninic acid assay
BCR: Biochemical recurrence
CCK-8: Cell counting kit 8
Co-IP: Coimmunoprecipitation
CRPC: Castration-resistant prostate cancer
DAPI: 4′,6-Diamidino-2-phenylindole dihydrochloride
DEGs: Differentially expressed genes
DKFZ: Deutsches Krebsforschungszentrum (The German Cancer Research Center)
DMEM: Dulbecco’s modified eagle medium
DMSO: Dimethyl sulfoxide
EdU: 5-Ethynyl-2′-deoxyuridine
FDR: False discovery rate
GEO: Gene expression omnibus
GSE: GEO series
GSEA: Gene set enrichment analysis
GST: Glutathione S-transferase
GTEx: Genotype-tissue expression project
HEK-293T: Human embryonic kidney cell 293T
IHC: Immunohistochemistry
IP: Immunoprecipitation
JNK: C-Jun N-terminal kinase
KEGG: Kyoto Encyclopedia of Genes and Genomes
MBL: Mannose/mannan-binding lectin
MSKCC: Memorial Sloan Kettering Cancer Center
NES: Normalized enrichment score
OD: Optical density
PCa: Prostate cancer
PCR: Polymerase chain reaction
PEI: Polyethyleneimine
PFS: Progression-free survival
PSA: Prostate specific antigen
RNA-Seq: RNA sequencing
RPMI: Roswell Park Memorial Institute
RRID: Research resource identifiers
RT-qPCR: Real-time quantitative PCR
SD: Standard deviations
SDS-PAGE: Sodium dodecyl sulfate polyacrylamide gel electrophoresis
shRNA: Small hairpin RNA
TCGA: The cancer genome atlas
TCGA-PRAD: The cancer genome atlas prostate adenocarcinoma
UPGMA: Unweighted pair group method with arithmetic mean
