# Supplementary material for: Aspartoacylase suppresses prostate cancer progression by blocking LYN activation
Source: Mil Med Res. 2023 Jun 5;10:25. doi: 10.1186/s40779-023-00460-0 (PMC10240701; doi:10.1186/s40779-023-00460-0)
Supplement: Supplementary file 1 — Additional file 1: Table S1. Characteristics of patients providing samples for RNA-Seq. Table S2. Characteristics of patients providing samples for Western blotting. Table S3. Characteristics of patients providing samples for IHC. Table S4. Characteristics of patients providing samples for RT-qPCR. Fig. S1. ASPA is down-regulated in PCa patients. Fig. S2. ASPA overexpression inhibits DU145 cell proliferation and migration in vitro. Fig. S3. ASPA knockdown promotes DU145 cell proliferation and migration in vitro. Fig. S4. ASPA negatively regulates JNK1/2-C-Jun activity in PC-3 cells. Fig. S5. LYN mediates the function of ASPA in PC-3 cells. [file 40779_2023_460_MOESM1_ESM.pdf]

Table S1 Patient characteristics for RNA-seq

Patient case (#)	Age (year)	Smoking history	BMI (kg/m^2)	Pre-operative PSA (ng/ml)	Gleason score	pT stage	N stage
Paired samples							
#1	70	No	20.2	37.539	9	T3b	N1
Unpaired samples							
Tumor tissues							
#1	72	No	22.2	29.82	8	T3	N0
#2	73	No	23.9	34.447	9	T2	N0
#3	66	No	NA	11.854	7	T2	N0
#4	74	No	25.4	31.072	8	T3a	N0
#5	67	No	20.8	9.189	7	T2	NA
#6	74	No	25.9	20.985	8	T3	N0
#7	57	No	22.9	15.193	7	T2	N0
#8	72	No	23.9	202.472	9	T3b	N1
#9	67	No	20.4	153.544	9	T3b	N0
#10	66	No	NA	11.854	7	T2	N0
#11	65	Yes	23.4	11.6	7	T2	N0
#12	72	No	29.1	13.48	7	T2	N0
#13	66	No	24.5	50.414	8	T3b	N0
#14	61	No	21.4	40	8	T3a	N0
#15	72	No	21.8	13.666	7	T2	N0
#16	73	No	NA	17.756	7	T2	NA
#17	62	Yes	27.8	15.644	7	T2	N0
Adjacent normal tissues							
#1	79	No	21.3	25.821	7	T2	N0
#2	78	No	25.8	27.141	7	T2c	N0
#3	65	No	18.9	6.49	7	T1a	N0
#4	78	No	21.4	30.2	8	T2	N0
#5	68	Yes	25	12.983	0	T3b	N1
#6	73	No	17.4	4.43	6	T1a	N0
#7	84	No	24.913	79.9	7	T2a	N0
#8	59	Yes	26.1	193.932	7	T3b	N0

BMI body mass index, *PSA* prostate specific antigen, *NA* not assessed, *pT* pathological T

Table S2 Characteristics of patients providing samples for Western blotting (paired samples)

Patient case (#)	Age (year)	Smoking history	BMI (kg/m^2)	Pre-operative PSA (ng/ml)	Gleason score	pT stage	N stage
#1	65	Yes	25.3	12.173	NA	T4	N0
#2	70	No	20.415	57.512	7	T2	N0
#3	57	No	22.8	12.8	7	NA	NA
#4	73	No	NA	17.756	7	T2	N0
#5	70	No	25.7	14.532	7	NA	NA
#6	71	No	21.9	326.746	9	T2	N0
#7	65	Yes	23.4	11.6	7	T2	N0
#8	67	No	22.6	22.114	7	T3a	N0
#9	71	No	21.9	9.63	8	T3a	N0
#10	60	Yes	20.8	16.353	7	T2	N0
#11	58	No	25.6	14.2	7	T2	N0
#12	68	Yes	26	1.589	7	T2	N0
#13	64	Yes	18.4	31.6	9	T2	N0
#14	73	No	23.4	7.641	7	T3a	N0
#15	82	No	24.4	9.597	9	T2	N0
#16	59	No	25.2	17.848	6	T3a	N0
#17	72	No	25.8	7.488	10	T2	N0
#18	60	Yes	25.8	17.5	7	T3a	N0

BMI body mass index, *PSA* prostate specific antigen, *NA* not assessed, *pT* pathological T

Table S3 Characteristics of patients providing samples for IHC (paired samples)

Patient case (#)	Age (year)	Smoking history	BMI (kg/m^2)	Pre-operative PSA (ng/ml)	Gleason score	pT stage	N stage
#1	71	No	21.9	9.63	8	T3a	N0
#2	64	Yes	18.4	31.6	9	T2	N0
#3	68	Yes	26	1.589	7	T2	N0
#4	59	No	25.2	17.848	6	T3a	N0
#5	65	Yes	23.4	11.6	7	T2	N0

IHC immunohistochemistry, *BMI* body mass index, *PSA* prostate specific antigen, *pT* pathological T

Table S4 Characteristics of patients providing samples for RT-qPCR

Patient case (#)	Age (year)	Smoking history	BMI (kg/m^2)	Pre-operative PSA (ng/ml)	Gleason score	pT stage	N stage
Paired samples							
#1	68	No	25.8	58	8	T2	N0
#2	77	No	22.3	10.378	7	T2a	N0
#3	78	No	21.4	30.2	8	T2	N0
#4	57	Yes	22.5	24.117	9	T3b	N1
#5	76	Yes	24.1	70.311	7	T2c	N0
#6	75	No	18	54.338	7	T2	N0
#7	70	No	20.2	37.539	9	T3b	N1
#8	79	No	21.3	25.821	7	T2	N0
#9	72	No	25.8	7.488	10	T2	N0
Unpaired samples							
Tumor tissues							
#1	74	No	25.9	20.985	8	T3	N0
#2	66	No	NA	11.854	7	T2	N0
#3	70	No	25.7	14.532	7	NA	NA
#4	61	No	21.4	40	8	T3a	N0
#5	67	No	20.4	153.544	9	T3b	N0
#6	72	No	29.1	13.48	7	T2	N0
#7	70	No	23.9	1.82	6	T2	N0
#8	69	No	26.298	9.223	8	T2	N0
#9	67	No	20.4	40.839	7	T2c	N0
#10	66	No	24.5	50.414	8	T3b	N0
#11	57	No	22.9	15.193	7	T2	N0
#12	76	No	27	19.024	9	T3b	N1
#13	57	No	23.4	12.731	8	T3a	N0
#14	72	No	22.2	29.82	8	T3	N0
#15	65	Yes	23.4	11.6	7	T2	N0
#16	63	Yes	25	48.549	7	T3b	N1
#17	72	No	21.8	13.666	7	T2	N0
Adjacent normal tissues							
#1	63	No	24.2	11.689	6	T2b	N0
#2	71	No	21.9	326.746	9	T2	N0
#3	68	Yes	25	12.983	10	T3b	N1
#4	61	Yes	23.7	9.205	9	T4	N1
#5	73	No	17.4	4.43	6	T1a	N0
#6	84	No	24.913	79.9	7	T2a	N0
#7	67	Yes	21.2	12.31	9	T2	N0
#8	74	No	23.7	0.245	9	T2	N0
#9	78	No	21.2	14.784	9	T3b	N1
#10	72	Yes	25.6	18.85	8	T3a	N0

#11	60	Yes	25.8	17.5	7	T3a	N0
#12	78	No	25.8	27.141	7	T2c	N0
#13	65	No	18.9	6.49	7	T1a	N0
#14	64	Yes	18.4	31.6	9	T2	N0

RT-qPCR real-time quantitative PCR, *BMI* body mass index, *PSA* prostate specific antigen, *NA* not assessed, *pT* pathological T

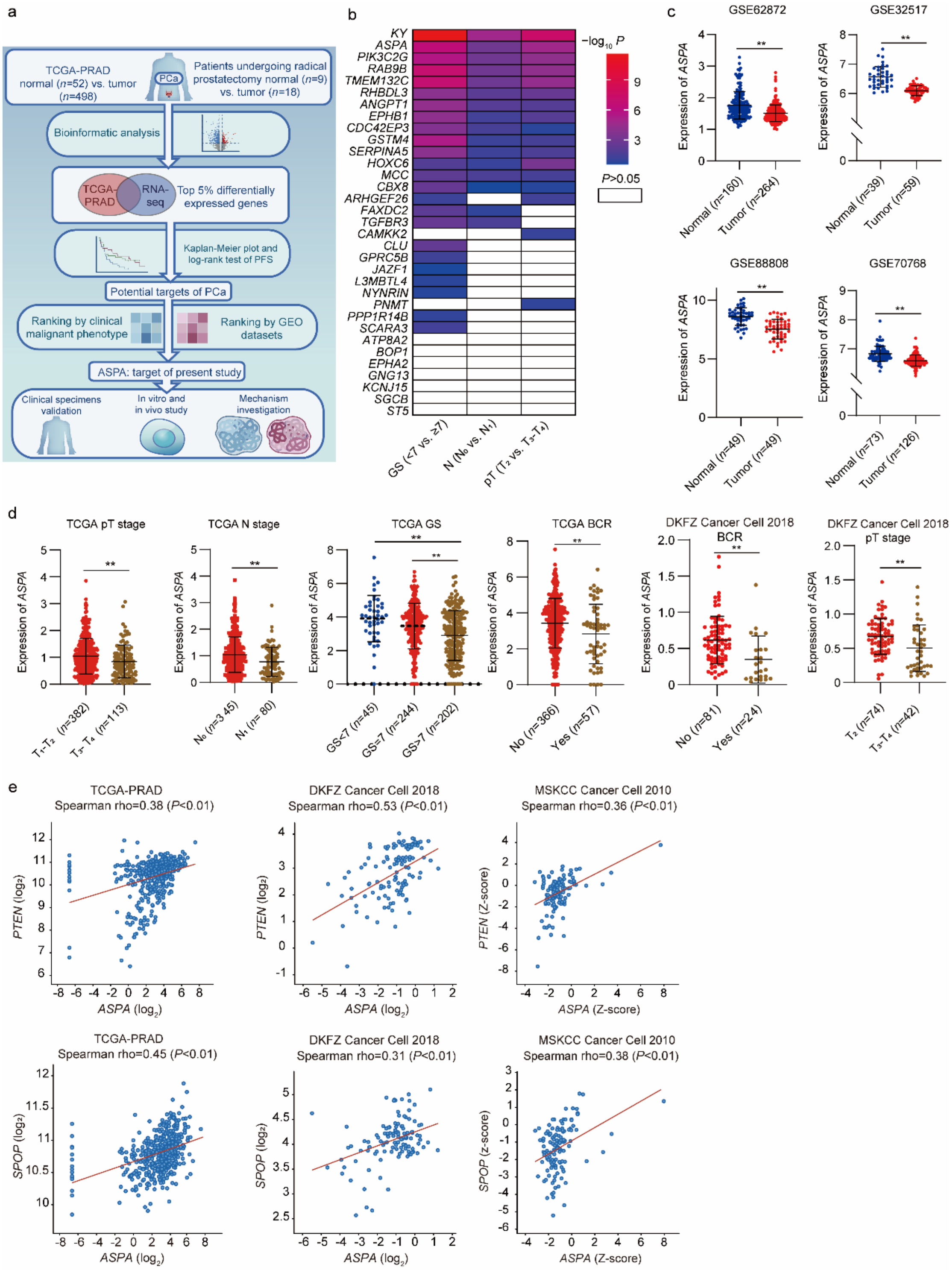

Fig. S1 ASPA is down-regulated in PCa patients. **a** The study design chart. **b** Heatmap analysis of PFS-related differentially expressed genes with GS, N stage, and pT stage in the TCGA-PRAD dataset. **c** The expression level of *ASPA* in the four GEO datasets. **d** The expression of *ASPA* stratified by pT stage, N stage, GS, and BCR in TCGA-PRAD dataset and DKFZ Cancer Cell 2018 dataset. **e** Correlationship between *ASPA* expression and *PTEN* or *SPOP* at mRNA levels in PCa tissues from TCGA-PRAD dataset (n = 491), DKFZ Cancer Cell 2018 dataset (n = 118), and MSKCC Cancer Cell 2010 dataset (n = 128). The data are presented as the mean ± standard deviation (SD). ASPA aspartoacylase, BCR biochemical recurrence, DKFZ Deutsches Krebsforschungszentrum, GEO gene expression omnibus, GS Gleason

score, GSE gene expression omnibus series, pT pathological T, PTEN phosphatase and tensin homolog, MSKCC Memorial Sloan Kettering Cancer Center, SPOP speckle type BTB/POZ protein, PCa prostate cancer, PFS Progression-free survival, TCGA-PRAD The Cancer Genome Atlas Prostate Adenocarcinoma. ** $P < 0.01$

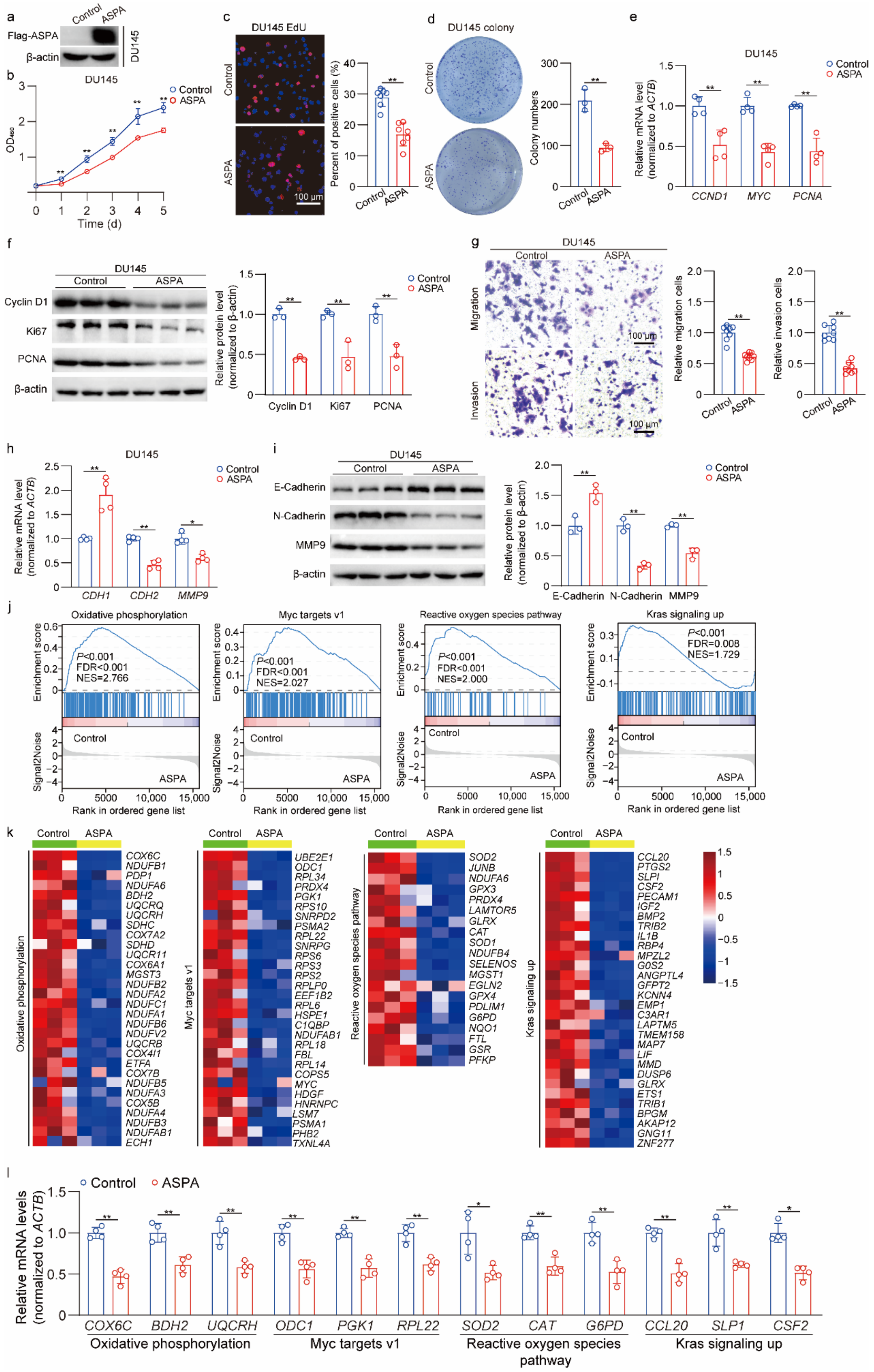

Fig. S2 ASPA overexpression inhibits DU145 cell proliferation and migration in vitro. **a** Western blotting

results of ASPA protein expression in DU145 cells transfected with control or ASPA overexpression vector. The protein expression was normalized to β-actin levels. **b** CCK-8 assay showed that ASPA overexpression inhibited DU145 cell proliferation. **c** Representative images of EdU-positive DU145 cells transfected with control or ASPA overexpression vector (scale bar = 100 μm). The graph on the right shows the percentage of EdU-positive nuclei. The data were obtained from 7 fields of 3 independent experiments. **d** Colony formation assay showed that ASPA overexpression inhibited DU145 cell colony formation ability. The graph on the right shows the colony numbers from 3 independent experiments. **e** RT-qPCR results of proliferation-related genes in DU145 cells transfected with control or ASPA overexpression vector. The mRNA expression was normalized to *ACTB* levels. **f** Western blotting results (left) and quantification results (right) of proliferation-related proteins in DU145 cells transfected with control or ASPA overexpression vector. The protein expression was normalized to β-actin levels. **g** Transwell assays showed that ASPA overexpression inhibited DU145 cell migration and invasion (scale bar = 100 μm). The graph on the right shows the relative migrating cells and the relative invading cells. The data were obtained from 8 fields of 3 independent experiments. **h** RT-qPCR results of epithelial-mesenchymal transition genes in DU145 cells transfected with control or ASPA overexpression vector. The mRNA expression was normalized to *ACTB* levels. **i** Western blotting results (left) and quantification results (right) of epithelial-mesenchymal transition proteins in DU145 cells transfected with control or ASPA overexpression vector. The protein expression was normalized to β-actin levels. **j** The GSEA enrichment plot of the top four significantly altered cancer hallmarks based on the RNA-seq datasets in PC-3 cells from the control group and ASPA overexpression group. **k** Heatmaps showed the significantly altered genes related to the top four altered cancer hallmarks based on the RNA-seq datasets from the control group and ASPA overexpression group in PC-3 cells. **l** The expression of genes related to the top four altered cancer hallmarks in PC-3 cells transfected with control or ASPA overexpression vector. The data are presented as the mean ± standard deviation (SD). ASPA aspartoacylase, CCK-8 cell counting kit 8, CCND1 cyclin D1, CDH1 cadherin 1, CDH2 cadherin 2, EdU 5-ethynyl-2'-deoxyuridine, FDR false discovery rate, GSEA gene set enrichment analysis, MYC v-Myc myelocytomatosis viral oncogene homolog, MMP9 matrix metallopeptidase 9, NES normalized enrichment score, OD optical density, PCNA proliferating cell nuclear antigen, RT-qPCR real-time quantitative PCR. * $P < 0.05$, ** $P < 0.01$

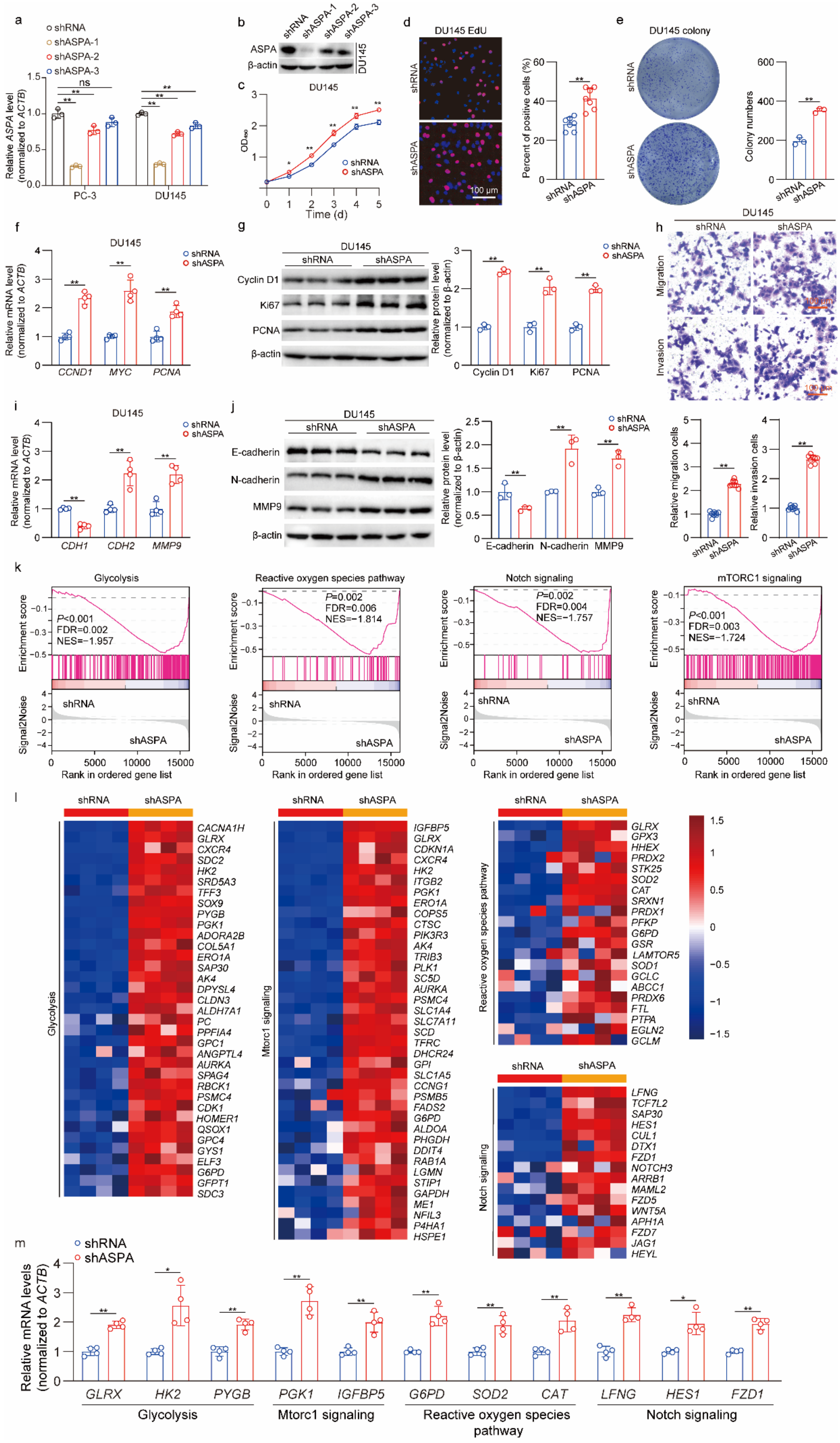
a
shRNA
shASPA-1
shASPA-2
shASPA-3
Relative ASPA level (normalized to ACTB)
PC-3
DU145
ns
b
shRNA
shASPA-1
shASPA-2
shASPA-3
ASPA
β-actin
DU145
c
DU145
OD450
Time (d)
shRNA
shASPA
d
DU145 EdU
shRNA
shASPA
100 μm
Percent of positive cells (%)
e
DU145 colony
Colony numbers
f
DU145
Relative mRNA level (normalized to ACTB)
CCND1
MYC
PCNA
g
DU145
Cyclin D1
Ki67
PCNA
β-actin
Relative protein level (normalized to β-actin)
h
DU145
Migration
Invasion
i
DU145
CDH1
CDH2
MMP9
j
DU145
E-cadherin
N-cadherin
MMP9
β-actin
Relative migration cells
Relative invasion cells
k
Glycolysis
P<0.001
FDR=0.002
NES=−1.957
Reactive oxygen species pathway
P=0.002
FDR=0.006
NES=−1.814
Notch signaling
P=0.002
FDR=0.004
NES=−1.757
mTORC1 signaling
P<0.001
FDR=0.003
NES=−1.724
Enrichment score
Signal2Noise
Rank in ordered gene list
l
Glycolysis
CACNA1H GLRX CXCR4 SDC2 HK2 SRD5A3 TFF3 SOX9 PYGB PGK1 ADORA2B COL5A1 ERO1A SAP30 AK4 DPYSL4 CLDN3 ALDH7A1 PC PPFIA4 GPC1 ANGPTL4 AURKA SPAG4 RBCK1 PSMC4 CDK1 HOMER1 QSOX1 GPC4 GYS1 ELF3 G6PD GFPT1 SDC3
Mtorc1 signaling
IGFBP5 GLRX CDKN1A CXCR4 HK2 ITGB2 PGK1 ERO1A COPS5 CTSC PIK3R3 AK4 TRIB3 PLK1 SC5D AURKA PSMC4 SLC1A4 SLC7A11 SCD TFRC DHCR24 GPI SLC1A5 CCNG1 PSMB5 FADS2 G6PD ALDOA PHGDH DDIT4 RAB1A LGMN STIP1 GAPDH ME1 NFIL3 P4HA1 HSPE1
Reactive oxygen species pathway
GLRX GPX3 HHEX PRDX2 STK25 SOD2 CAT SRXN1 PRDX1 PFKP G6PD GSR LAMTOR5 SOD1 GCLC ABCC1 PRDX6 FTL PTPA EGLN2 GCLM
Notch signaling
LFNG TCF7L2 SAP30 HES1 CUL1 DTX1 FZD1 NOTCH3 ARRB1 MAML2 FZD5 WNT5A APH1A FZD7 JAG1 HEYL
m
Relative mRNA levels (normalized to ACTB)
GLRX HK2 PYGB PGK1 IGFBP5 G6PD SOD2 CAT LFNG HES1 FZD1
Glycolysis
Mtorc1 signaling
Reactive oxygen species pathway
Notch signaling

Fig. S3 *ASPA* knockdown promotes DU145 cell proliferation and migration in vitro. **a** RT-qPCR results showed the mRNA expression of *ASPA* in PC-3 cells (left) and DU145 cells (right) transfected with shRNA or shASPA. The mRNA expression was normalized to *ACTB* levels. **b** Western blotting results of ASPA protein expression in DU145 cells transfected with shRNA or shASPA. The protein expression was normalized to β-actin levels. **c** CCK-8 assay showed that *ASPA* knockdown promoted DU145 cell proliferation. **d** Representative images of EdU-positive DU145 cells transfected with shRNA or shASPA (scale bar = 100 μm). The graph on the right shows the percentage of EdU-positive nuclei. The data were obtained from 7 fields of 3 independent experiments. **e** Colony formation assay showed that *ASPA* knockdown promoted DU145 cell colony formation ability. The graph on the right shows the colony numbers from 3 independent experiments. **f** RT-qPCR results of proliferation-related genes in DU145 cells transfected with shRNA or shASPA. The mRNA expression was normalized to *ACTB* levels. **g** Western blotting results (left) and quantification results (right) of proliferation-related proteins in DU145 cells transfected with shRNA or shASPA. The protein expression was normalized to β-actin levels. **h** Transwell assays showed that *ASPA* knockdown promoted DU145 cell migration and invasion (scale bar = 100 μm). The graph below shows the quantification results for Transwell assays. The data were obtained from 8 fields of 3 independent experiments. **i** RT-qPCR results of epithelial-mesenchymal transition genes in DU145 cells transfected with shRNA or shASPA. The mRNA expression was normalized to *ACTB* levels. **j** Western blotting results (left) and quantification results (right) of epithelial-mesenchymal transition proteins in DU145 cells transfected with shRNA or shASPA. The protein expression was normalized to β-actin levels. **k** GSEA enrichment plot of the top four significantly altered cancer hallmarks except top one based on the RNA-seq datasets in PC-3 cells from the shRNA group and shASPA group. **l** Heatmaps showed the significantly altered genes related to the top four altered cancer hallmarks based on the RNA-seq datasets from shRNA group and shASPA group in PC-3 cells. **m** The expression of genes related to the top four altered cancer hallmarks in PC-3 cells transfected with shRNA or shASPA. The data are presented as the mean ± standard deviation (SD). ASPA aspartoacylase, CCK-8 cell counting kit 8, CCND1 cyclin D1, CDH1 Cadherin 1, CDH2 Cadherin 2, EdU 5-ethynyl-2'-deoxyuridine, FDR false discovery rate, GSEA gene set enrichment analysis, MYC v-Myc myelocytomatosis viral oncogene homolog, MMP9 matrix metallopeptidase 9, NES normalized enrichment score, OD optical density, PCNA proliferating cell nuclear antigen, RT-qPCR real-time quantitative PCR, shRNA small hairpin RNA. $^{*}P < 0.05$, $^{**}P < 0.01$

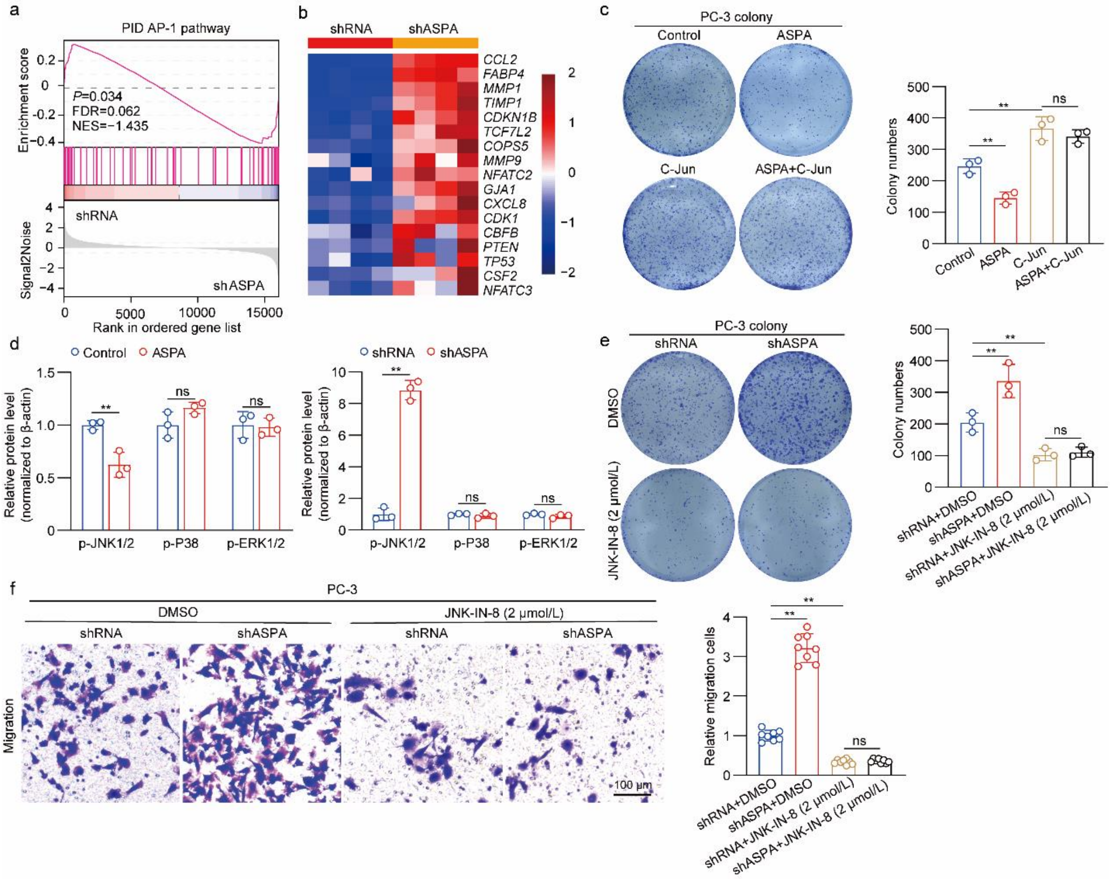

Fig. S4 ASPA negatively regulates JNK1/2-C-Jun activity. **a** Enrichment of the AP-1 pathway in the shRNA group and shASPA group and analysis by GSEA based on RNA-seq datasets in PC-3 cells. **b** Heatmap showed the significantly altered genes related to the AP-1 pathway based on the RNA-seq datasets in PC-3 cells transfected with shRNA or shASPA. **c** The cell colony formation ability of PC-3 cells cotransfected with ASPA overexpression vector and/or C-Jun overexpression vector was assessed using a colony formation assay. The graph on the right shows the colony numbers from 3 independent experiments. **d** The quantification results of Western blotting related to **Fig. 5i**. The protein expression was normalized to β-actin levels. **e** Cell colony formation ability of PC-3 cells transfected with shASPA and/or treated with JNK-IN-8 was assessed using a colony formation assay. The graph on the right shows the colony numbers from 3 independent experiments. **f** The cell migration ability of PC-3 cells transfected with shASPA and/or treated with JNK-IN-8 was assessed using a transwell assay. The graph on the right shows the relative migration of cells. The data were obtained from 8 fields of 3 independent experiments (scale bar = 100 μm). The data are presented as the mean ± standard deviation (SD). ASPA aspartoacylase, AP-1 activator protein-1, C-Jun v-Jun avian sarcoma virus 17 oncogene homolog, DMSO dimethyl sulfoxide, ERK extracellular regulated protein kinases, GSEA gene set enrichment analysis, FDR false discovery rate, JNK c-Jun N-terminal kinase, NES normalized enrichment score, shRNA small hairpin RNA, PID pathway interaction database. ** $P < 0.01$, ns not significant

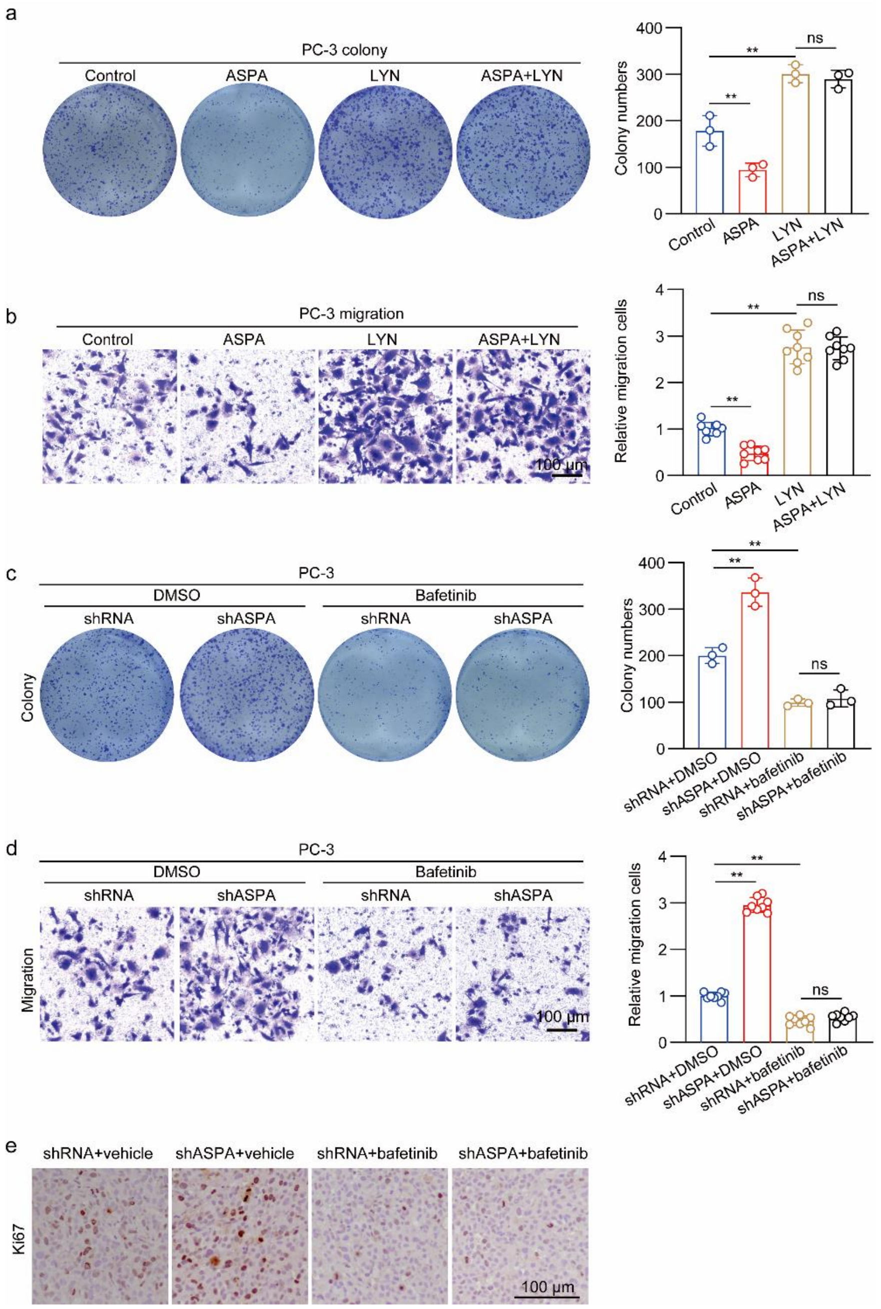

Fig. S5 LYN mediates the function of ASPA in PCa cells. **a** Cell colony formation ability of PC-3 cells cotransfected with ASPA overexpression vector and/or LYN overexpression vector were assessed using colony formation assays. The graph on the right shows the colony numbers from 3 independent experiments. **b** Cell migration ability of PC-3 cells cotransfected with ASPA overexpression vector and/or LYN overexpression vector were assessed using Transwell assays. The graph on the right shows the relative migrated cells obtained from 8 fields of 3 independent experiments (scale bar = 100 μm). **c** Cell colony formation ability of PC-3 cells transfected with shASPA and/or treated with bafetinib were assessed using colony formation assays. The graph on the right shows the colony numbers from 3 independent experiments. **d** Cell migration ability of PC-3 cells transfected with shASPA and/or treated with bafetinib were assessed using Transwell assays. The graph on the right shows the relative migrated cells obtained from 8 fields of 3 independent experiments (scale bar = 100 μm). **e** Representative images

of IHC showed the expression of Ki67 in xenograft tumors derived from PC-3 cells transfected with shASPA and/or treated with bafetinib (scale bar = 100 μm). The data are presented as the mean ± standard deviation (SD). ASPA aspartoacylase, DMSO dimethyl sulfoxide, LYN Lck/Yes-related novel protein tyrosine kinase, shRNA small hairpin RNA. ** $P < 0.01$, ns not significant
